# Growth rate-dependent mutations and phenotypic switching facilitate rapid adaptation

**DOI:** 10.1038/s41540-026-00758-4

**Published:** 2026-06-16

**Authors:** Anton Grishechkin, Omer Karin

**Affiliations:** Department of Mathematics, Imperial College London, London, UK

**Keywords:** Biophysics, Computational biology and bioinformatics, Ecology, Ecology, Evolution, Microbiology, Systems biology

## Abstract

The mutation rates of many species are regulated in response to internal and external conditions. Studies in several model organisms have shown that mutation rates can be modulated by the organism’s growth rate, particularly through stress-induced mutagenesis. Here, we investigate the functional consequences of this coupling. Using a model of asexual evolution on a high-dimensional, single-peaked fitness landscape, we show that regulating mutation rate through growth rate provides an efficient strategy for rapid adaptation. This mechanism maximizes growth by balancing mutation and selection pressures. We approximate the optimal coupling as a biphasic response function that prescribes a low, constant mutation rate at high growth rates, which gradually increases once growth falls below a critical threshold. This adaptive strategy depends on two key parameters: mutational gain and critical growth rate, both determined by environmental conditions. This raises the question of how organisms establish these parameters in fluctuating environments. We demonstrate that weak selection in well-adapted populations, combined with long-term phenotypic fluctuations, can generate variability in these parameters. Such standing variation prepares populations for rapid evolution following environmental change. Our findings connect this adaptive strategy to specific molecular regulatory circuits and provide predictions for sequential antibiotic treatment regimens.

## Introduction

Mutations provide the raw material for evolution, and mutation rates themselves are often under biological control. In organisms from bacteria like E. coli to eukaryotes like yeast, mutation rate is not a fixed quantity but is rather a plastic trait, often dependent on both environmental and internal signals^1–10^.

In E. coli, this mutational plasticity has been studied extensively, particularly in the context of stress-induced mutagenesis^1,11–13^. Growth rate appears to be one of the key determinants of the mutation rate. Bacteria under slow growth conditions, such as in stationary phase or following antibiotic exposure, often exhibit elevated mutation rates^14,15^. Moreover, different environmental stresses, such as distinct nutrient limitations, can increase mutation rate^16–18^. If a beneficial mutation allows an adapted strain to resume faster growth, its mutation rate typically reverts to a lower baseline level^19^. However, whether the control of mutation rates by growth rates is itself an adaptation shaped by direct selection, or if it is a by-product of cellular mechanisms involved in stress responses remains a topic of debate^20^.

Focusing on E. coli, at the molecular level, mutation rates are mediated by several growth sensitive pathways which are often interlinked^1,21–24^. One of the main pathways, which is up-regulated in a wide variety of growth-limiting environments is the general stress response pathway^22,25–27^ governed by the alternative sigma factor RpoS^5,21,28^. RpoS activity, which increases during slow growth^29^, has been linked to elevated mutation rates through downregulation of DNA mismatch repair and upregulation of the error-prone DNA polymerase Pol IV^30–34^. An additional link between RpoS and growth rate is established through the stringent response and its alarmone, (p)ppGpp^35^. (p)ppGpp is a key regulator of growth rate control^36–38^, and promotes RpoS accumulation^39–42^, further supporting the model of a growth-dependent regulation of mutation rates.

While the existence of growth-dependent mutation rates is well established experimentally, their functional significance remains debated^20^. We hypothesised that, given the widespread occurrence of mutational plasticity across diverse organisms and the variety of pathways that elevate mutation rates, growth-dependent regulation could represent an optimal strategy for adapting to new environments. Previous theoretical work suggested that mutation rates should be high when fitness is low and decrease as fitness approaches an optimum^43–49^. Moreover, models incorporating phenotypic plasticity in mutation rates have shown that variability of responses within a population plays an important role in ensuring successful adaptation in fluctuating environments^50–52^. Experimental observations further indicate that hypermutation typically arises only within a subpopulation^13,19,53,54^.

Here, we investigate how the regulation of mutation rates by growth rates can facilitate adaptation to novel environments. To this end, we develop a mathematical framework based on Fisher’s Geometric Model (FGM) of adaptation in an asexual population evolving on a high-dimensional phenotypic landscape^55–57^. This framework allows us to analyse how feedback between growth and mutation rates can optimize adaptive dynamics. We find that when all individuals share the same growth rate, the optimal mutation response is biphasic: mutation rates remain low at high growth rates but increase once growth drops below a critical threshold. This response depends on two environmentally determined parameters—a critical growth rate and a mutational gain. When growth rates are heterogeneous, however, the biphasic response becomes suboptimal at high growth rates, as maintaining low but nonzero mutation rates can promote further adaptation.

Crucially, we show that an efficient adaptive response is highly sensitive to environmental parameters, which are likely to vary during environmental change. We propose that organisms can overcome the limitations of the biphasic response by generating long-term fluctuations in the growth-mutation response parameters. These fluctuations create standing variation in adapted populations that can be rapidly exploited following an environmental shift. Moreover, variability in response parameters can mitigate the suboptimality of the biphasic response at high growth rates, thereby enabling continued adaptation. Our theory provides a quantitative foundation for understanding stress-induced mutagenesis and offers insights into strategies for controlling the evolution of pathogenic organisms.

## Results

### Dimensionless model for adaptation

We model the evolution of an asexual population of fixed size *N*. In our framework, we define an organism’s fitness as its growth rate, *μ*. We use a Wright-Fisher model, where an individual’s probability of reproduction is proportional to its growth rate (Methods)^58^.

To connect phenotype to fitness, we utilise the framework of FGM^55^. An organism’s phenotype is represented by a vector of $\widehat{n}$ quantitative traits, $x\in {{\mathbb{R}}}^{\widehat{n}}$. In a given environment, we assume there is a single optimal phenotype, x^*^, which confers the maximum possible growth rate, ${\mu }_{\max }$. The growth rate of an organism with a suboptimal phenotype *x* declines according to a Gaussian function of its distance from the optimum:

$$\mu (x)={\mu }_{\max }{e}^{-\frac{1}{2}{(x-{x}^{* })}^{T}S(x-{x}^{* })}$$

Here, the ‘selection matrix’ *S* describes how strongly each trait is selected for and how the traits interact^59^. Mutations cause random displacements in this phenotypic space, with the number of mutations acquired per generation having mean, *ρ* (mutations per generation), and the effect size and direction of mutations determined by a covariance matrix *M*.

We approximate mutational effects as uniformly distributed displacements on a hypersphere (Methods). In this simplified model, an organism’s state can be described by a single scalar: its dimensionless phenotypic distance, *d*, from the optimum. The growth rate function then simplifies to:

1 $$\mu (d)={\mu }_{\max }{e}^{-\frac{n}{4}{d}^{2}}.$$

In this scaled form, *n* is the effective number of phenotypic dimensions under selection (the rank of the matrix *S*) and is an environmentally dependent quantity in our model.

Similarly, the combined effects of mutation rate and selection strength are captured by a single dimensionless mutation radius, *r*. This parameter connects back to the physical parameters via the key scaling relation:

2 $$r=\sqrt{2\beta \rho }.$$

Here, *ρ* is the per generation per individual mutation rate and *β* is a scalar measuring the strength of selection (derived from the matrix *S*, Methods). Our analysis proceeds by studying the evolution of the distance *d* under the influence of mutations of radius *r*, providing general analytical results that can be mapped back to specific biological settings using these scaling relations.

### A biphasic mutation strategy resolves the speed-accuracy trade-off

**Fig. 1:**
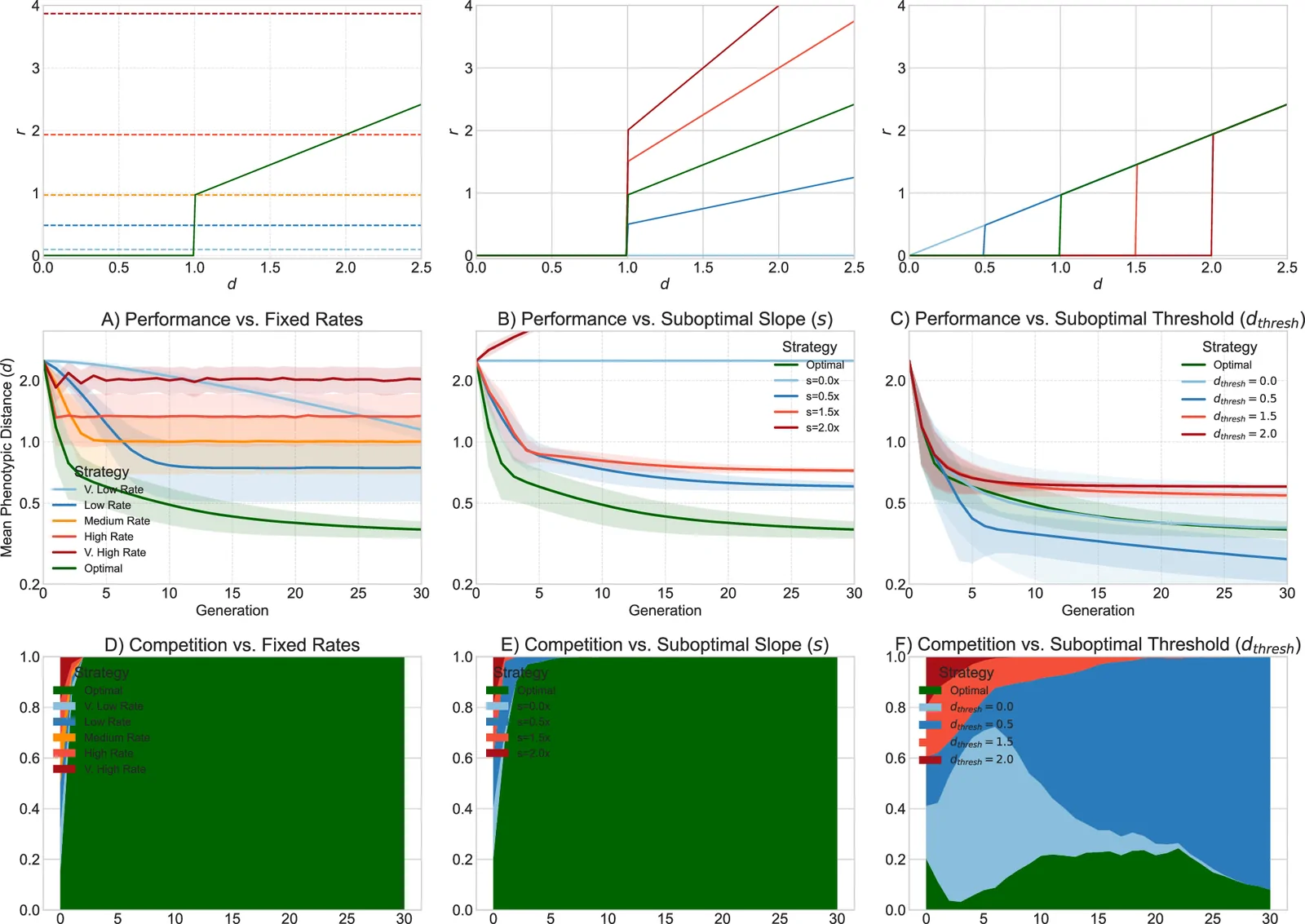
Simulation of biphasic growth-dependent adaptation strategy. The panels at the top show the strategy maps used in simulations below, plotting the dimensionless mutation radius (r) against phenotypic distance (d). **A–C** Performance of various mutational strategies over 30 generations, starting from a phenotypic distance *d* = 2.5. Bold lines represent the mean of 50 replicate simulations, with the faint area being ± 1 standard deviation. The *y*-axis is on a logarithmic scale to highlight differences in equilibrium distance. **A** The estimated optimal biphasic strategy (dark green) achieves the rapid initial adaptation of high fixed-rate strategies (reds/orange) while maintaining the low equilibrium mutational load of low fixed-rate strategies (blues). **B** Compared to strategies with suboptimal slopes (s), **C** Compared to strategies with different activation threshold *d*_thresh_, **D–F** Direct competition between the strategies shown in the corresponding top panels. Stack plots show the proportion of each strategy in the population over time. Simulation parameters: *N* = 10000, *n* = 5.

A fixed mutation rate imposes a fundamental constraint on adaptation. While a high mutation rate promotes rapid evolution in a poorly-adapted population, it also increases mutational load by the accumulation of deleterious mutations, preventing the population from attaining higher fitness. We analysed this trade-off by considering the dynamics of a population in a single-peaked fitness landscape (Methods). We first estimate the mutation-selection balance in this setting, d^*^, which is the equilibrium distance from the optimum, under the assumption of no variance in phenotypic distance from the peak within the population. In this case, d^*^ is proportional to the mutation radius *r*, with the relationship given by ${d}^{* }\approx \frac{r}{2{K}_{n,N}}$, where *K*_*n*,*N*_ is an environmentally determined constant (with the approximation being valid for large *r*, see Methods). This direct relationship illustrates that a fixed-rate strategy cannot be simultaneously fast and accurate (Fig. 1A).

This trade-off can be resolved by a plastic strategy where the mutation rate is modulated by the organism’s distance from the optimum, *d* (see also ref. ^60^). In our model, this distance directly reflects the growth rate, *μ*, providing an internally measurable proxy for the degree of maladaptation. To derive a plastic mutational strategy, we optimised the expected rate of adaptation at each generation, under the assumption that the population has the same phenotypic distance from the optimum at each generation. This assumption corresponds to a scenario dominated by hard selective sweeps^61^, where each generation descends from a single successful ancestor. We found that the optimal strategy under this assumption is biphasic (Methods), that is, it has two distinct regimes of response.

In this biphasic strategy, when the population is poorly adapted and far from the optimum (*d* ≫ 1), the optimal mutation radius, *r*_opt_ scales linearly with this distance, *r*_opt_(*d*) ≈ *K*_*n*,*N*_*d*. The term *K*_*n*,*N*_ is a dimensionless environmental constant that quantifies the efficacy of selection. It integrates the effects of the number of traits under selection (*n*) and the population size (*N*), conceptually representing the ease of adaptation in a given environment (adaptation becomes harder the more traits are under selection, Methods).

**Fig. 2:**
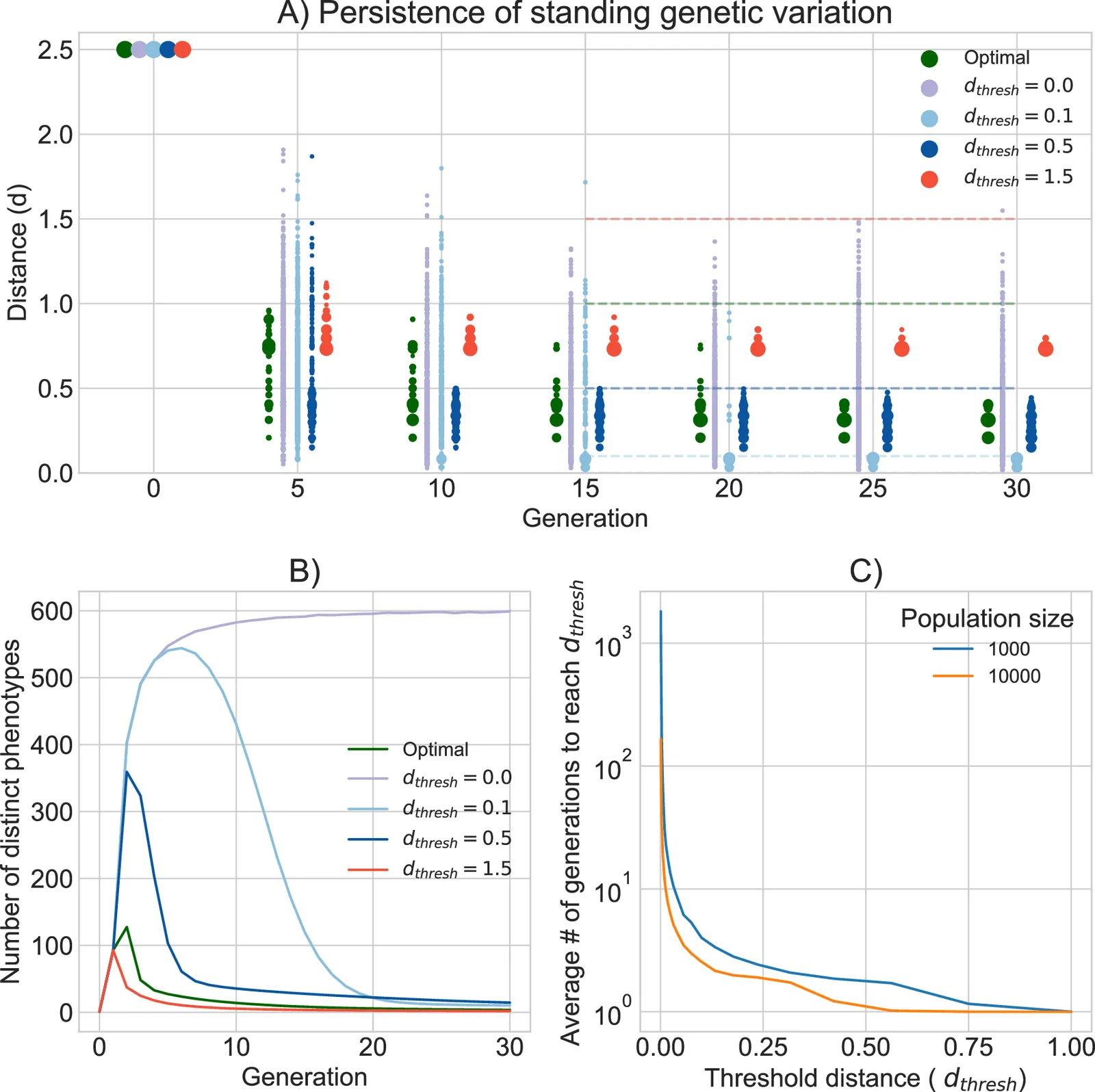
Standing genetic variation promotes adaptation for biphasic strategies in the weak selection regime. **A** For a population employing a biphasic strategy, once all individuals fall below the threshold, *d*_thresh_, selection is the only force acting on the population. Each point represents a distinct phenotype, with the vertical position giving the distance to the optimum *d* (y-axis), while the radius of the point it represents is scaled as ${r}_{i}\propto \sqrt{{N}_{i}}$, where *N*_*i*_ is the number of individuals sharing that phenotype. **B** The number of distinct phenotypes present in the population over time for a given threshold strategy. **C** The mean number of generations required for a phenotype with *d* < *d*_thresh_ to first appear, shown for different population sizes. Simulation parameters: *N* = 1000 (**A**, **B**), *n* = 5 and populations start with an initial distance to the peak, *d*_0_ = 2.5.

Conversely, when the population is well-adapted (*d* ≪ 1), the optimal strategy under our assumption minimises the mutation rate to preserve high fitness, *r*_opt_(*d*) ≈ 0 (Fig. 1A). This regime corresponds to weak selection, where our assumption of negligible standing variation becomes less reliable. Standing genetic variation emerges rapidly, as with neutral selection one expects to have, in equilibrium, *N*(1 − 1/*e*) distinct phenotypes (Fig. 2B, Methods).

Adaptation in a population with individuals employing the biphasic strategy typically progresses rapidly through the *d* ≫ 1 regime, resulting in an approach to *d* ≈ 1 within a few generations (Fig. 1A). Importantly, the strategy is highly sensitive to the precise gain in the response in the strong selection regime (*d* ≫ 1). That is, when we consider a general response:

3 $$r(d)=\left\{\begin{array}{ll}sd & d > {d}_{{\rm{thresh}}}\\ 0, & d\le {d}_{{\rm{thresh}}},\end{array}\right.$$

we find strong sensitivity to the gain parameter *s* in the *d* ≫ 1 regime (Fig. 1B). However, there is much weaker dependence of the adaptation strategy on the critical distance *d*_thresh_ (Fig. 1C), which we examine below. Taken together, these results demonstrate that biphasic control of mutation rate enables both rapid and accurate adaptation. The optimal biphasic response depends on two environment-specific quantities, the mutational gain and the threshold distance.

In the weak selection regime (*d* < 1), standing genetic variation accumulates, and for such a heterogeneous population a strict step response at *d* = 1 is no longer optimal (Figs. 1, S1). Under a threshold strategy, phenotypes with *d* < *d*_thresh_ do not mutate, so once the entire population falls below *d*_thresh_, selection is the only force acting on the population; eventually, the best available phenotype dominates the population (Fig. 2A,B). Before this point is reached, however, the population splits into two subgroups: individuals below the threshold, whose phenotypes are effectively frozen, and individuals above it, who continue to mutate and may occasionally discover a phenotype with a higher growth rate than any currently present.

The choice of *d*_thresh_ therefore involves a compromise. Lowering *d*_thresh_ brings the final population closer to the peak (Fig. 2A), but the time required for an individual to mutate to a phenotype below the threshold grows exponentially as *d*_thresh_ → 0 (Fig. 2C). In particular, a purely linear strategy (*r*(*d*) = *Cd* for all *d*, with no threshold) will always underperform, since there is zero probability of a mutation landing exactly at the peak (Fig. 1C,F). A non-zero threshold is thus preferable, though its optimal value need not coincide with *d* = 1 and will depend on the relative importance of speed and accuracy in a given setting. We describe one strategy that can further improve adaptation in this regime in the Methods (Methods, Fig. S3), noting that this strategy would require a population-level coordination between individuals.

### Rapid adaptation to novel environments requires precise tuning of response parameters

Since cells can sense their growth rate *μ* but not their distance *d*, we translated the biphasic strategy into a biologically implementable form. The mutation rate corresponding to the biphasic strategy, *ρ*_bph_, as a function of growth rate is characterized by two measurable parameters: a critical growth rate (*κ*) below which mutagenesis is initiated, and a mutational gain (*α*) that governs the strength of the response. The resulting control function can be captured by (Methods):

4 $${\rho }_{{\rm{bph}}}(\mu )\approx \alpha \log \left(\frac{{\mu }_{\max }}{\mu }\right){{\mathcal{S}}}_{\kappa }\left(\mu \right),$$

where ${{\mathcal{S}}}_{\kappa }\left(\mu \right)$ is a decrea sing sigmoidal response function that interpolates between a minimal value of ${{\mathcal{S}}}_{\kappa }\left(\mu \right)=0$ for $\mu ={\mu }_{\max }$ and a maximal value of ${{\mathcal{S}}}_{\kappa }\left(\mu \right)=1$ at *μ* < *κ*, with *κ* corresponding to the threshold *d* = 1 via the scaling $\kappa \approx {\mu }_{\max }{e}^{-\frac{n}{4}}$. Similarly, the mutational gain, corresponding to the strategy having *r* ≈ *K*_*n*,*N*_*d* for *d* ≫ 1, is given by $\alpha =\frac{2{K}_{n,N}^{2}}{n\beta }$.

**Fig. 3:**
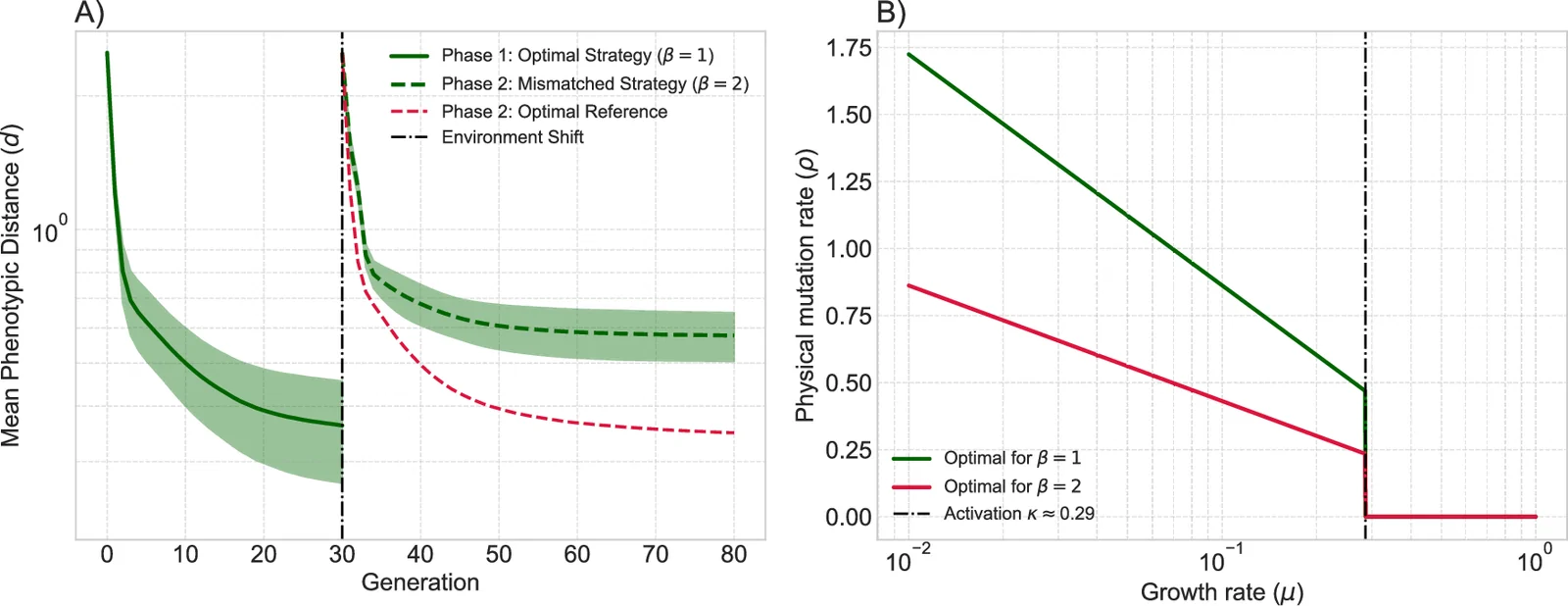
A strategy optimized for one environment is mismatched and maladaptive following a shift in selection strength (*β*). **A** Simulation of adaptation dynamics before and after an environmental shift. In Phase 1 (Generations 0-30), a population adapts optimally in an environment with selection strength *β* = 1.0 (solid green line). At generation 30, the environment shifts to one with stronger selection, *β* = 2.0. The dashed green line shows the subsequent trajectory of the population, which continues to use the strategy optimized for the previous environment. This now-mismatched strategy leads to slow adaptation and a high equilibrium mutational load. The dashed crimson line serves as a reference, showing the rapid adaptation that would be achieved by a strategy perfectly optimized for the new *β* = 2.0 environment. Shaded areas show ± 1 standard deviation from the mean and bold lines show the mean (*N* = 5000, *n* = 5). **B** The underlying optimal physical mutation rate (*ρ*) as a function of growth rate (*μ*) for the two environments. The green curve shows the optimal strategy for the initial, weaker selection environment (*β* = 1.0), while the crimson curve shows the optimal strategy for the stronger selection environment (*β* = 2.0). Both strategies activate at the same critical growth rate, but the required mutational gain is substantially higher when selection is weaker. The mismatch shown in (**A**) occurs because the population continues to operate on the green curve after the environment has shifted, resulting in a mutation rate that is too high for the new selective conditions.

The optimal values of the mutational gain, *α*, and the critical growth rate, *κ*, are highly sensitive to the environmental parameters (*n*, *β*, *N*). We demonstrate this in Fig. 3A,B, where we show how a given scaled optimal response function, given by Eq. (3), may translate into markedly different optimal growth-response functions given by Eq. (4) under different environmental conditions. Therefore, a regulatory strategy optimized for one environment is unlikely to be effective in a novel one.

**Fig. 4:**
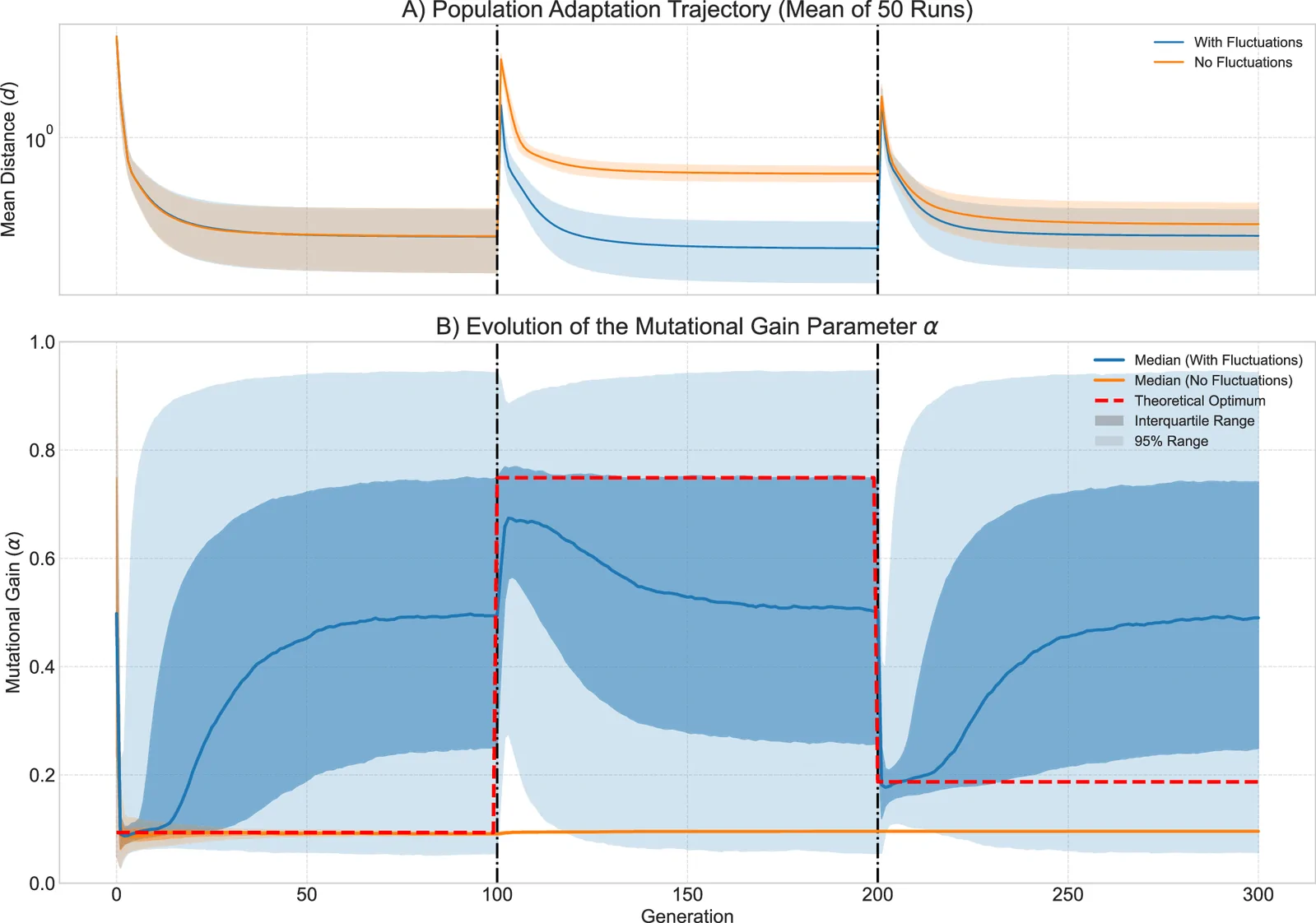
Phenotypic fluctuations maintain strategic diversity and facilitate rapid adaptation to novel environments. This figure compares the evolutionary dynamics of two populations over 300 generations, averaged over 50 independent simulations. Both populations evolve in a fluctuating environment that shifts at generations 100 and 200, with the selection strength changing from *β* = 4.0 (left) to *β* = 0.5 (centre) to *β* = 2.0 (right). One population experiences ongoing phenotypic fluctuations in its strategy parameters (blue), while the other does not (orange). **A** The mean population adaptation trajectory. The y-axis shows the mean phenotypic distance (*d*) from the optimum. The population with fluctuations consistently re-adapts faster following both environmental shifts. Shaded areas represent one standard deviation across the 50 simulation runs. **B** The evolution of the mutational gain parameter, *α*. This panel reveals the mechanistic basis for the difference in adaptability. The median evolved *α* for each population is shown as a solid line, with layered shading representing the interquartile (darker shade) and 95% (lighter shade) ranges of the population’s diversity. The red dashed line indicates the theoretical optimum for each environment. The population without fluctuations suffers a rapid collapse in its strategic diversity after adapting to the first environment, leaving it unable to evolve towards the new optima in subsequent phases. In contrast, the fluctuating population maintains a broad range of standing variation, which allows selection to act efficiently and drive the median *α* towards the new optimum after each shift. Simulation parameters: Population size *N* = 5000, phenotypic dimensions *n* = 5, fluctuation probability *p* = 0.05, and initial distance *d*_0_ = 2.5 after each environmental shift. Initially both populations started with varied mutational gain, with *α*_*i*_ ~ Unif(0, 1). In the population with fluctuations, the mutational gain had a probability *p* = 0.05 of switching per individual per generation. If an individual switches its phenotype, the mutational gain was resampled uniformly from (0, 1) (note 0≤*K*_*n*,*N*_≤1).

To probe the selective forces acting on these parameters, we simulated a population with initial heterogeneity in *α*, adapting to multiple distinct environments, with the environments being switched over time (Fig. 4, non-fluctuating strategy). Here, the environments differ in their selection strengths, *β*. We assume that the individuals are poorly adapted to the new environment at the point of the environmental switch. This scenario is consistent both with environments that select on different phenotypic trait combinations (such as in refs. ^62,63^) and with environments that select on the same traits but have optima at sufficiently separated locations in phenotype space (such as in ref. ^64^).

When the population was introduced to a new environment to which it was poorly adapted, individuals with mutational gains close to the new optimal one (i.e., close to $2{K}_{n,N}^{2}/(n\beta )$) adapted significantly faster. Consequently, strong selection rapidly fixed the optimal strategy, leading to a swift collapse in the population’s variation within just a few generations.

When both heterogeneity in *α* and *κ* are considered, we found that selection on the critical growth rate is much weaker than on the mutational gain, because the biphasic strategy below the threshold is largely insensitive to the exact critical value (Figs. S2, S4).

We hypothesized that the rapid optimisation of the mutational gain, and thus a collapse in variability of the parameters, would create a vulnerability to subsequent environmental shifts. To test this, we subjected the now-adapted, parameter-homogeneous population to a second environmental change with different properties. The population’s previously optimal strategy was now severely mismatched to the new challenge. As a result, adaptation was impeded, with the mismatched mutational gain leading to a sluggish evolutionary response (Fig. 4). Selection on the response parameters for the present environment, therefore, acts at the expense of the variation required for future challenges.

### Phenotypic fluctuations facilitate further adaptation in the weak selection regime and allow long-term evolvability

We identified two key conditions under which the biphasic strategy may fail to promote adaptation. First, the strong dependence of adaptation on specific response parameters poses a challenge in fluctuating environments, as parameters that are optimal in one context are unlikely to remain advantageous after subsequent environmental changes. Second, in the weak selection regime, population variability provides an adaptive advantage that the strategy does not exploit.

We propose that both limitations of the biphasic response can be effectively mitigated through multi-generational, non-genetic fluctuations in response parameters, which we will refer to throughout as phenotypic fluctuations. Biological stress responses, such as the general stress response, often exhibit non-genetic fluctuations. As examples in E. coli, sigma factors such as RpoS exhibit fluctuations over time^65,66^, while Ada, a DNA repair enzyme, exhibits stochastic variation from cell to cell, and thus cells with low Ada protein abundance exhibit higher mutagenesis^67^.

The key mechanism that can be utilised by these fluctuations is the state-dependent nature of the selective pressures on the mutational gain, *α*, and the critical growth rate, *κ*. For clarity, we focus here on fluctuations in the mutational gain *α*; fluctuations in the critical growth rate *κ* can independently aid adaptation, including in the weak selection regime, and are examined separately in the Supplementary and Methods (Fig. S2, Methods).

As dictated by the biphasic response function (Eq. (4)), selection on these parameters is strong only in maladapted, slow-growing populations. Once a population is well-adapted and growing near its maximum rate, the mutagenic response is suppressed, rendering the precise values of *α* nearly selectively neutral. This period of relaxed selection provides a window of opportunity for phenotypic switching (for example, via epigenetic modifications or noisy gene expression) to restore heterogeneity in the control parameters throughout the population without incurring a significant fitness cost (Fig. 4A,B). We also note that fluctuations in *κ* may also improve flexibility in the weak selection regime, helping compensate for the biphasic strategy’s limitations in that context (Fig. S2, Methods).

We demonstrated this by simulating populations in environments that changed periodically (every *τ* = 100 generations, Figs. 4A,B,S2). At each environmental switch, we assume the population is poorly adapted to the new environment. A population capable of phenotypic switching (where individual *α* values were reset with a small probability *p* each generation) consistently outperformed one with fixed, heritable parameters. The simulations confirm that during periods of stability, phenotypic switching regenerates substantial standing variation in the control parameters at minimal fitness cost. This variation is then rapidly exploited by selection following an environmental shift, enabling swift re-adaptation. In this way, the population leverages periods of higher growth to accumulate variation that can then be used to adapt to subsequent environmental shifts.

## Discussion

Here, we identify a growth-mutation coupling that resolves the fundamental speed-accuracy trade-off in adaptation. The response we derive depends on environment-specific parameters, yet its underlying form reflects general aspects of the mutation-selection process. We show that this challenge of environmental specificity can be overcome by multi-generational phenotypic fluctuations, which allow a population to maintain the variation required for rapid adaptation to novel conditions.

Previous theoretical explorations of fitness-dependent mutation have generally considered either purely discrete shifts in response to stress^46,60^ or strictly gradual changes^43,46,49,60^. By analysing adaptation within a high-dimensional phenotypic landscape, our model unifies these concepts, revealing that conditioned on no variation within the population, optimal control of mutation rate is biphasic with a switch-like increase at an intermediate growth rate, followed by a graded response as growth continues to decline. In deriving the biphasic strategy, we did not assume any specific biological mechanism linking growth and mutation rates. Such coupling could arise either from direct regulatory control of mutagenesis or indirectly from growth-dependent constraints on repair capacity, such as reduced mismatch repair under resource limitation.

Our model reveals a unique advantage inherent to growth-dependent regulation. The selective pressure on the regulatory parameters themselves is state-dependent. When a population is well-adapted and growing fast, the mutagenic response is silenced, rendering the precise values of the control parameters nearly selectively neutral. This creates a “safe harbour" where phenotypic fluctuations can regenerate variation without a fitness cost. This feature, which is absent in fixed mutation rate strategies, is critical for maintaining long-term evolvability, allowing a population to prepare for future challenges during times of high growth.

The biphasic strategy rests on two empirically supported principles. First, mutation rates rise as growth rates decline. Second, the coupling factors underlying this relationship are subject to non-heritable fluctuations.

The first principle is well-established across taxa. In E. coli, the RpoS general stress response and the (p)ppGpp stringent response provide molecular pathways linking slow growth to elevated mutagenesis^30–33^. Comparable links exist in eukaryotes, where the expression of stress-response genes in yeast correlates more strongly with growth rate than with the particular stressor applied^68^, and these pathways are known to influence mutation rates^2,69^. Our model suggests that by positioning mutagenesis downstream of such a general growth-rate-sensing system, organisms can implement a near-optimal adaptive strategy.

This coupling gives the strategy’s two parameters, the mutational gain *α* and the critical growth rate *κ*, concrete mechanistic interpretations. A useful decomposition writes the mutation rate as *ρ* = *ρ*_*b*_(1 − *p*_rep_), separating error production from repair. The dependence of *ρ*_*b*_ on growth rate differs by source. Damage from exogenous or metabolic insults accumulates per unit time, so that *ρ*_*b*_ ∝ 1/*μ* per generation and *α* scales naturally with lesion rate. Replication-associated errors, by contrast, occur per generation, and an increase in *α* at low growth rates would instead require active changes in error production or repair. In both cases, *κ* reflects a threshold below which repair efficiency declines sharply, for example, through RpoS-mediated downregulation of mismatch repair or growth-rate-dependent induction of the SOS response. In the Supplementary, we also provide biological interpretations of other model parameters (Supplementary Table 1).

The second principle, that response parameters fluctuate non-heritably, is consistent with multi-generational variation in RpoS expression^65,66^, stochastic variation in Ada protein levels^67^, and the observation that elevated mutation rates typically arise in only a subpopulation of cells^53^. These fluctuations are likely under long-term selection and may be lost when not needed, consistent with the attenuation of elevated mutation rates in mutator lineages during prolonged adaptation to a single environment^70^. Our framework is pathway-agnostic and can accommodate any combination of regulatory circuits satisfying these two principles, including cases where mutagenesis is a by-product of stress-response machinery rather than a directly selected trait, though optimal control need not be achieved in that scenario.

Our model highlights the role of variation in driving adaptation under weak selection (high-growth) conditions, echoing the distinction between hard and soft selective sweeps^61,71^. In a *hard sweep*, a new beneficial mutation arises and rapidly fixes, erasing most linked genetic variation, whereas *soft sweeps* draw on pre-existing or recurrent mutations, allowing multiple genetic backgrounds to contribute to adaptation. From this perspective, the biphasic strategy can be viewed as tuning the mutation rate to maximise the likelihood of hard sweeps when selection is strong, while maintaining variability that supports soft sweeps under weak selection.

Since our derivation of the biphasic response as an optimal mechanism assumed no variation, it is plausible that organisms could improve their adaptation rates if they were able to estimate population-level variation in growth rates. Consider, for example, a mixed population containing slower-growing organisms with *d* ≈ 1 and faster-growing organisms with *d* ≪ 1. In such a case, suppressing mutation rates in the slower-growing organisms would be highly disadvantageous for them, as they would be rapidly outcompeted by the faster-growing ones. In fact, in the presence of such variability, these organisms experience much stronger selective pressure than in the no-variability setting. Consequently, adopting a riskier strategy of higher mutation rates becomes more beneficial, as it may provide at least some chance of overcoming the faster-growing competitors (See also Methods).

The proposed model potentially accounts for the observed variation in mutation rate elevation across environments^16,17^. Organisms with a biphasic response exhibit environment-specific behaviour because the key parameters, the critical growth rate and the mutational gain, depend on environmental conditions. For example, when the critical growth rate is very low, most organisms show little or no increase in mutation rate, whereas differences in the optimal mutational gain can explain the varying fold increases observed under different environmental conditions.

The dynamic interplay between the collapse and regeneration of the regulatory variation has direct implications for clinical strategies aimed at combating microbial evolution and resistance to antibiotics. Our model predicts that after a pathogen population adapts to a first drug, the variation in its adaptive machinery will be temporarily depleted. Administering a second, mechanistically distinct antibiotic (different *β*, *n*) before this variation has had time to regenerate could be a particularly effective strategy. By exploiting this transient period of reduced evolvability, it may be possible to hinder the population’s ability to mount an effective response to the subsequent treatment.

## Methods

### Model details

To simulate evolution in discrete generations, we adopt a Wright-Fisher framework in which reproductive success is weighted by growth rate. The fitness of an organism in our model is its Malthusian growth rate, *μ*. This instantaneous rate *μ* (units of time^−1^) is linked to the cell division time, where we assume cells have a doubling time, *T*_*d*_ (units of time). The relationship is given by:

$$\mu =\frac{\log (2)}{{T}_{d}}$$

We model a population of fixed size *N* using a discrete-generation Wright-Fisher (WF) framework^58^. In each generation, *N* offspring are sampled with replacement from the parent generation. The probability *p*_*j*_ of selecting parent *j* is proportional to its growth rate *μ*_*j*_ when adjusted by the average population growth rate:

5 $${p}_{j}=\frac{{\mu }_{j}}{{\sum }_{k=1}^{N}{\mu }_{k}}.$$

This provides a discrete-generation approximation to selection under continuous exponential growth. Equivalently, this sampling rule arises from a Gillespie-type algorithm in which the next reproducing organism is chosen with probability proportional to its growth rate.

We next specify how phenotype determines growth rate, using Fisher’s Geometric Model (FGM) to capture stabilizing selection toward a single environment-specific optimum^55^. An organism’s phenotype is a vector $x\in {{\mathbb{R}}}^{\widehat{n}}$, where $\widehat{n}$ is the number of quantitative traits. In a given environment, we assume that there is a single optimal phenotype, x^*^, that maximizes the growth rate to ${\mu }_{\max }$. We further assume that the growth rate function *μ*(*x*) is a multivariate Gaussian centred at x^*^:

$$\mu (x)={\mu }_{\max }{e}^{-\frac{1}{2}{(x-{x}^{* })}^{T}S(x-{x}^{* })}.$$

Here, *S* is a positive semidefinite matrix describing the strength of stabilizing selection on the traits^59^. *S* being positive semidefinite means some combination of phenotypic traits are allowed to be selectively neutral, namely the ones that correspond to the 0 eigenvectors of *S*.

Mutations cause random displacements in this phenotypic space. A single mutation $\widehat{m}$ is drawn from a multivariate Gaussian distribution, $\widehat{m} \sim {\mathcal{N}}(0,M)$, where *M* is the mutation covariance matrix, which for simplicity we assume to be strictly positive definite. We assume that the number of mutations an individual accumulates per generation, *H*, follows a Poisson distribution, *H* ~ Poi(*ρ*), where *ρ* is the mutation rate per individual per generation. Thus, before selection, daughter-cell phenotypes will be given by

$${x}^{M}=x+\mathop{\sum }\limits_{i=1}^{H}{\widehat{m}}_{i},$$

where ${\widehat{m}}_{i}$ are i.i.d random variables from ${\mathcal{N}}(0,M)$ distribution.

For analytical tractability, we next perform a series of transformations. We first rotate and rescale the phenotypic space so that selection acts isotropically and only on traits that contribute to fitness. We shift the coordinate system to set the optimal phenotype x^*^ = 0. We then perform a linear transformation on the phenotype space to factorise the selection effects. This transforms the phenotype *x* into a new set of coordinates $y\in {{\mathbb{R}}}^{n}$, where *n* = rank(*S*) is the effective dimensionality of the phenotypic space of the current environment. The growth rate in this new space depends only on the squared Euclidean distance from the origin:

$$\mu (y)={\mu }_{\max }{e}^{-\beta \frac{1}{2}{y}^{T}y}={\mu }_{\max }{e}^{-\beta \frac{1}{2}| | y| {| }^{2}}$$

where *β* is a scalar capturing the overall selection strength.

Our coordinate transformation consists of the following. As *S* is positive semidefinite, we can always decompose it via a Cholesky decomposition, namely *S* = *βA*^*T*^*A*, where *A* is an upper triangular matrix and *β* is the maximal eigenvalue of *S* (to see that *β* also corresponds to the selection strength see Methods section below). Further if ${\rm{rank}}(A) < \widehat{n}$, then by a relabelling of the coordinates, we can always ensure *A* has the following block form:

6 $$A=\left(\begin{array}{cc}{A}_{1} & {A}_{2}\\ 0 & 0\\ \end{array}\right),$$

where *A*_1_ is a *n* × *n* upper-triangular matrix, where *n* = rank(*A*). Thus by rescaling the model so that $\widetilde{y}=A(x-{x}^{* })$, we get that our growth function is now given by

$$\mu (\widetilde{y})={\mu }_{\max }\exp \left(-\frac{\beta }{2}{\widetilde{y}}^{T}\widetilde{y}\right).$$

This also gives that the phenotype of the organisms after the next division is given by

$${\widetilde{y}}^{M}=\widetilde{y}+A\mathop{\sum }\limits_{i=1}^{H}{\widehat{m}}_{i}.$$

Note then that as *A* is of the form given by Eq.(6), we have that ${\widetilde{y}}_{k}=0,{\widetilde{y}}_{k}^{M}=0$ for *k* > *n* and so we take $y\in {{\mathbb{R}}}^{n}$ to be the vector of the first *n* components of $\widetilde{y}$. Thus making our growth rate be given by

$$\mu (y)={\mu }_{\max }\exp (-\frac{\beta }{2}| | y| {| }^{2}),$$

and the phenotype after division being

$${y}^{M}=y+\mathop{\sum }\limits_{i=1}^{H}{m}_{i},$$

where *m*_*i*_ are now given by ${m}_{i} \sim {\mathcal{N}}(0,\widetilde{A}\widetilde{M}{\widetilde{A}}^{T})$ and $\widetilde{A}$ is now $n\times \widehat{n}$, i.e., $\widetilde{A}$ has the following block form:

7 $$\widetilde{A}=\left(\begin{array}{cc}{A}_{1} & {A}_{2}\\ \end{array}\right).$$

Note that this transforms the phenotypic space into a coordinate frame where the basis vectors are linearly independent and orthogonal, which corresponds to the case of idealised phenotypic traits. Moreover, it removes the traits which do not contribute to the growth rate.

To make the mutational process analytically tractable, we introduce one assumption and one approximation. First, we assume mutations are isotropic in the transformed space^57^, meaning they have no preferred direction. Second, we approximate the stochastic sum of Poisson-distributed mutations by a single mutational step of a fixed, effective radius, $\widehat{r}$, in a uniformly random direction, chosen to preserve the expected squared displacement. The mutational radius $\widehat{r}$ is related to the underlying mutation rate *ρ* via the expected squared mutational effect size, such that ${\widehat{r}}^{2} \sim \rho n$.

Utilising once again the Cholesky decomposition, we write *M* as *M* = *LL*^*T*^, where *L* is lower-triangular. We get then that by setting *G* = *AL*, we have

$${y}^{M}=y+G\mathop{\sum }\limits_{i=1}^{H}{\zeta }_{i},$$

where ${\zeta }_{i} \sim \mathrm{i.i.d}\,\,{\mathcal{N}}(0,{I}_{n})$. We set

$$Y:=\mathop{\sum }\limits_{i=1}^{H}{\zeta }_{i},$$

as our compound Poisson distribution vector. We have that as *ζ*_*i*_ are radially symmetric, so is *Y*, as the sum of independent radially symmetric random variables is radially symmetric. Therefore we may write *Y* = *RU*, where *U* ~ Unif(*S*^*n*−1^), *S*^*n*−1^ is the unit hypersphere in ${{\mathbb{R}}}^{n}$ and *R* is the random radial part of *Y*. Note that

$${R}^{2}=| | \mathop{\sum }\limits_{i=1}^{H}{\zeta }_{i}| {| }^{2}.$$

To the authors’ best knowledge there is no known characterisation for the distribution of *R*^2^. As such instead here we derive the expectation and the variance of *R*^2^. By the tower law, we may write

8 $${\mathbb{E}}[f({R}^{2})]={\mathbb{E}}\left[{\mathbb{E}}\left[f\left(| | \mathop{\sum }\limits_{i=1}^{H}{\zeta }_{i}| {| }^{2}\right)| H\right]\right].$$

Note that when *H* is fixed, we have ${\sum }_{i=1}^{H}{\zeta }_{i} \sim {\mathcal{N}}(0,H{I}_{n})=d\sqrt{H}{\mathcal{N}}(0,{I}_{n})$. As the radial part of a normalised multivariate normal in *n* dimension follows a *χ*^2^(*n*) distribution, we get from Eq.(8) that

$${\mathbb{E}}[f({R}^{2})]={\mathbb{E}}[{\mathbb{E}}[f(H{\chi }^{2})| H]],$$

where *χ*^2^ ~ *χ*^2^(*n*). As such we have that the expectation is

$${\mathbb{E}}[{R}^{2}]={\mathbb{E}}[{\mathbb{E}}[H{\chi }^{2}| H]]={\mathbb{E}}[Hn]=\rho n.$$

Similarly we have

$${\mathbb{E}}[{R}^{4}]={\mathbb{E}}[{\mathbb{E}}[{H}^{2}{\chi }^{4}| H]]={\mathbb{E}}[{H}^{2}({n}^{2}+2n)]=({\rho }^{2}+\rho )({n}^{2}+2n).$$

Therefore the variance is

$${\rm{Var}}({R}^{2})=({\rho }^{2}+\rho )({n}^{2}+2n)-{\rho }^{2}{n}^{2}=\rho n(2\rho +n+2).$$

Our two simplifying assumptions then correspond to taking $R=\widehat{r}$ to be a constant, noting that if we match the second-order moments we get ${\widehat{r}}^{2}=\rho n$, and taking *G* = *I*, which is equivalent to mutations being isotropic in the idealised phenotypes. Thus, our mutated phenotype will be given in our setting by ${y}^{M}=y+\widehat{r}U$.

Finally, we rescale distance and mutational radius to absorb *β* and *n* into a single dimensionless mutation-selection parameter, yielding the form of the model used in the main analysis. We define a scaled phenotype $z:=(\sqrt{2\beta /n})y$ (Note the close correspondence of this scaling and the classical Fisher x-scale^55^) and the dimensionless distance as *d* ≔ ∣∣*z*∣∣. With this scaling, the growth rate function takes its final form:

$$\mu (d)={\mu }_{\max }{e}^{-\frac{n}{4}{d}^{2}}.$$

The mutational process is similarly rescaled. The dimensionless mutational radius *r* becomes $r=(\sqrt{2\beta /n})\widehat{r}$. Since $\widehat{r}=\sqrt{\rho n}$, this leads to the central scaling relation

$$r=\sqrt{2\beta \rho }.$$

The full dynamics are thus captured by the evolution of the dimensionless distance *d* under the influence of mutations of dimensionless radius *r*.

### Dynamics of adaptation

Here we provide the mathematical derivation for the dynamics of adaptation. Throughout this section, when it is clear from context, we use *d*(*t*) and *d* interchangeably for ease of legibility.

We begin by analysing the evolutionary dynamics for a population with a fixed, dimensionless mutation radius, *r*. To proceed with a mathematical analysis we assume that the population at generation *t* has no phenotypic variation, namely all individuals are a distance *d*(*t*) = : *d*, from the optimal phenotype.

The expected squared distance in the next generation, ${\mathbb{E}}[{d}^{2}(t+1)| d(t)]$, given the distance in the current generation *d*(*t*), is given by:

9 $${\mathbb{E}}[{d}^{2}(t+1)| d(t)]=d{(t)}^{2}+{r}^{2}-2rd(t){f}_{n,N}(r,d(t))$$

Here, the term *r*^2^ represents the dispersive effect of mutations, while the function *f*_*n*,*N*_ captures the efficacy of selection in directing evolution towards the optimum. This function depends on the phenotypic dimension *n*, the population size *N*, and the current state (*d*, *r*). We analyse this equation in two key limits:

- **Strong Selection (*d*** **≫ 1):** When the population is far from the optimum, selection is highly effective. It is also the limit in which our monomorphic-population assumption is most accurate, as each generation after selection will be composed of phenotypically identical individuals. In this limit, Eq. (9) simplifies to:
  10 $${\mathbb{E}}[{d}^{2}(t+1)| d(t)]\approx {d}^{2}(t)+{r}^{2}-2{K}_{n,N}rd(t)$$
  The term *K*_*n*,*N*_ is a dimensionless **environmental constant** that depends on *n* and *N*. It represents the expected progress towards the optimum for a given mutational step when selection is maximally efficient.
- **Weak Selection (*d*** **≪ 1):** When the population is near the optimum, selection is weak. Here, the equation becomes:
  11 $${\mathbb{E}}[{d}^{2}(t+1)| d(t)]\approx {d}^{2}(t)+{r}^{2}(1-{d}^{2}(t))$$
  Since *d* ≪ 1, the term *r*^2^(1 − *d*^2^(*t*)) is positive for any *r* > 0, indicating that mutation tends to push the population away from the optimum in this regime.

To derive Eq. (9) we proceed as follows. Assume that all individuals at generation *t* start at the same distance to the optimal phenotype, *d*(*t*) = : *d*. We are interested in the expected squared distance to the peak of the next generation, namely ${\mathbb{E}}[{d}^{2}(t+1)| d(t)]$. By the Tower law we can write it as

$${\mathbb{E}}[{d}^{2}(t+1)| d(t)]={\mathbb{E}}[{\mathbb{E}}[{d}^{2}(t+1)| {d}_{1}^{M}(t),...,{d}_{N}^{M}(t)]| d(t)],$$

where ${d}_{i}^{M}=| | {z}_{i}^{M}| |$, is the distance after mutation of individual i to the peak. By summing over all the potential mutated candidates for the next generation, we have

$${\mathbb{E}}[{d}^{2}(t+1)| d(t)]={\mathbb{E}}[\mathop{\sum }\limits_{i=1}^{N}\frac{{({d}_{i}^{M}(t))}^{2}\mu ({d}_{i}^{M}(t))}{{\sum }_{k=1}^{N}\mu ({d}_{k}^{M}(t))}| d(t)],$$

and thus we have

$${\mathbb{E}}[{d}^{2}(t+1)| d(t)]={\mathbb{E}}\left[\mathop{\sum }\limits_{i=1}^{N}\frac{{({d}_{i}^{M}(t))}^{2}\exp (-n{({d}_{i}^{M}(t))}^{2}/4)}{{\sum }_{k=1}^{N}\exp (-n{({d}_{k}^{M}(t))}^{2}/4)}| d(t)\right].$$

Now the key is to understand the properties of *d*^*M*^(*t*). We proceed by noting that

$${({d}_{i}^{M})}^{2}=| | {z}_{i}^{M}| {| }^{2}=| | z+r{U}_{i}| {| }^{2}=| | z| {| }^{2}+{r}^{2}+2r\langle z,{U}_{i}\rangle ={d}^{2}+{r}^{2}+2rd\langle s,{U}_{i}\rangle ,$$

where *s* is the directional unit vector pointing from *z* to 0. Plugging this back we get

$${\mathbb{E}}[{d}^{2}(t+1)| d(t)]={d}^{2}+{r}^{2}+2rd{\mathbb{E}}\left[\mathop{\sum }\limits_{i=1}^{N}\frac{\langle s,{U}_{i}\rangle \exp (-n({d}^{2}+{r}^{2}+2rd\langle s,{U}_{i}\rangle )/4)}{{\sum }_{k=1}^{N}\exp (-n({d}^{2}+{r}^{2}+2rd\langle s,{U}_{k}\rangle )/4)}| d\right].$$

Cancelling out we get:

$${\mathbb{E}}[{d}^{2}(t+1)| d(t)]={d}^{2}+{r}^{2}+2rd{\mathbb{E}}\left[\mathop{\sum }\limits_{i=1}^{N}\frac{\langle s,{U}_{i}\rangle \exp (-nrd\langle s,{U}_{i}\rangle /2)}{{\sum }_{k=1}^{N}\exp (-nrd\langle s,{U}_{k}\rangle /2)}| d\right].$$

The crucial trick now is to note that *U*_*i*_ is a spherically symmetric distribution, which means that for any unit vectors, *x*, *y* we have ${\mathbb{E}}[f(\langle x,{U}_{i}\rangle )]={\mathbb{E}}[f(\langle y,{U}_{i}\rangle )]$. Thus without loss of generality we may take *s* = *e*_1_, the first vector of the canonical basis. Letting *u*_*i*_ be the first coordinate of *U*_*i*_, we thus have 〈*s*, *U*_*i*_〉 = *u*_*i*_. Furthermore the distribution of the first coordinate of a uniform distribution over the hyper-sphere can be written as *u*_*i*_ = 2*B*_*i*_ − 1, where *B*_*i*_ is a beta distributed random variable with *B*_*i*_ ~ Beta((*n* − 1)/2, (*n* − 1)/2). As such our formula for the expected distance of the next generation can be further written as

$${\mathbb{E}}[{d}^{2}(t+1)| d(t)]={d}^{2}+{r}^{2}+2rd{\mathbb{E}}\left[\mathop{\sum }\limits_{i=1}^{N}\frac{(2{B}_{i}-1)\exp (-nrd(2{B}_{i}-1)/2)}{{\sum }_{k=1}^{N}\exp (-nrd(2{B}_{k}-1)/2)}| d\right],$$

and so simplifying we arrive at

12 $${\mathbb{E}}[{d}^{2}(t+1)| d(t)]={d}^{2}+{r}^{2}-2rd\left(1-2{\mathbb{E}}\left[\mathop{\sum }\limits_{i=1}^{N}\frac{{B}_{i}\exp (-nrd{B}_{i})}{{\sum }_{k=1}^{N}\exp (-nrd{B}_{k})}| d\right]\right).$$

Note that this is precisely Eq.(9), with *f*_*n*,*N*_ given by

$${f}_{n,N}(r,d(t))=1-2{\mathbb{E}}\left[\mathop{\sum }\limits_{i=1}^{N}\frac{{B}_{i}\exp (-nrd(t){B}_{i})}{{\sum }_{k=1}^{N}\exp (-nrd(t){B}_{k})}\right].$$

We look at the weak selection regime first. Namely we assume *d*(*t*), *r* ≪ 1 (Note that any *r* > 2*d* will always take any individual away from the peak and so if we care only about the optimal *r*, we may safely assume $r={\mathcal{O}}(d)$). We proceed via a Taylor expansion. Starting from Eq. (12) we get that

$${\mathbb{E}}[{d}^{2}(t+1)| d(t)]={d}^{2}+{r}^{2}-2rd\left(1-2{\mathbb{E}}\left[\mathop{\sum }\limits_{i=1}^{N}\frac{{B}_{i}(1-nrd{B}_{i})+{\mathcal{O}}({d}^{2})}{{\sum }_{k=1}^{N}(1-nrd{B}_{k}+{\mathcal{O}}({d}^{2}))}| d\right]\right),$$

and therefore

$${\mathbb{E}}[{d}^{2}(t+1)| d(t)]={d}^{2}+{r}^{2}-2rd\left(1-\frac{2}{N}\mathop{\sum }\limits_{i=1}^{N}{\mathbb{E}}\left[{B}_{i}-nrd{B}_{i}^{2}+\frac{nrd{B}_{i}}{N}{\sum }_{k=1}^{N}{B}_{k}+{\mathcal{O}}({d}^{2})\right]\right).$$

Now recall that *B*_*i*_ ~ Beta((*n* − 1)/2, (*n* − 1)/2) are identically distributed Beta random variables. Routine calculations give us that ${\mathbb{E}}[{B}_{i}]=1/2$, ${\mathbb{E}}[{B}_{i}^{2}]=(n+1)/(4n)$. As such we get

$${\mathbb{E}}[{d}^{2}(t+1)| d(t)]={d}^{2}+{r}^{2}-2rd\left[-\frac{nrd(n+1)}{4n}+\frac{nrd(n+1)}{4nN}+\frac{nrd(N-1)}{4N}+{\mathcal{O}}({d}^{2})\right].$$

Expanding and simplifying this yields

$${\mathbb{E}}[{d}^{2}(t+1)| d(t)]={d}^{2}+{r}^{2}-{r}^{2}{d}^{2}\frac{(N-1)}{N}+{\mathcal{O}}({d}^{3}).$$

Thus under the assumption of mildly large population size, so that we can take (*N* − 1)/*N* ≈ 1, we get that close to the peak, i.e., *d*(*t*) ≪ 1, our population progresses according to Eq. (11),

$${\mathbb{E}}[{d}^{2}(t+1)| d(t)]={d}^{2}(t)+{r}^{2}\left(1-{d}^{2}(t)\right).$$

Next we will consider, how our population adapts in the strong selection regime. Our starting point is Eq. (12). The strong selection regime occurs when *d*(*t*) ≫ 1 and it is useful to introduce the notion of an order statistic. In brief given our phenotypic distances *d*_1_, . . . , *d*_*N*_ we can order them in an ascending order labelled *d*_(1)_, . . . , *d*_(*N*)_, where *d*_(1)_ ≤ *d*_(2)_ ≤. . . ≤ *d*_(*N*)_. In this notation it is clear that the individual with the highest growth rate is a distance *d*_(1)_ away from the optimal phenotype. Also note that as our phenotypic space is ${{\mathbb{R}}}^{n}$, the probability that two individuals have the same phenotypic distance to the optimum after mutation is 0 (given *n* > 1). Note also that in this regime, the assumption that all the distances at the start of each generation are the same i.e., *d*_1_(*t*) = . . . = *d*_*N*_(*t*) is always valid.

So observe that in Eq. (12) the term inside the expectation,

$$\frac{\mathrm{exp}(-nrd(t){B}_{i})}{{\sum }_{k=1}^{N}\exp (-nrd(t){B}_{k})},$$

is in fact the same as the softmax function. We do not need any particular properties of the softmax, except note that as *d*(*t*) → *∞*, this softmax will behave like the function selecting the maximal argument of a vector,

$$\frac{\mathrm{exp}(-nrd(t){B}_{i})}{{\sum }_{k=1}^{N}\exp (-nrd(t){B}_{k})}\to 1,$$

if *B*_*i*_ < *B*_*k*_ for all *k* ≠ *i*. And otherwise

$$\frac{\exp (-nrd(t){B}_{i})}{{\sum }_{k=1}^{N}\exp (-nrd(t){B}_{k})}\to 0.$$

Note that this is precisely how one expects strong selection to behave, i.e., the individual with the highest growth rate always gets selected. As such in the strong selection regime Eq. (12) transforms into

$${\mathbb{E}}[{d}^{2}(t+1)| d(t)]={d}^{2}(t)+{r}^{2}-2rd(t)\left(1-2{\mathbb{E}}[{B}_{(1)}]\right).$$

One can interpret this as follows. Our mutations take us on a random hypersphere around the current phenotype. 1 − 2*B*_*i*_ is the difference the mutation makes in the direction of the phenotypic optimum (with more positive values getting us closer to the peak). As such we want to minimise the negative part, i.e., *B*_*i*_. Strong selection then precisely allows us to pick the individual with the highest value of 1 − 2*B*_*i*_, and thus the individual closest to the phenotypic optimum. Utilising standard results about order statistics and integration by parts, we can write the expectation of the beta variable as

$${\mathbb{E}}[{B}_{(1)}]={\int }_{0}^{1}{I}_{x}{(\frac{n-1}{2},\frac{n-1}{2})}^{N}dx,$$

where *I*_*x*_(*α*, *β*) is the regularised incomplete beta functions respectively. Note to derive this we have also utilised the symmetry of our beta random variables, namely *B* =*d*1 − *B*, as the beta distribution parameters are equal in our setting. Although for specific values of *N* = 2 and *N* = 3 one can solve this explicitly, while for a general *N* we managed to get asymptotic behaviours (See Methods below). As such we set ${K}_{n,N}=1-2{\mathbb{E}}[{B}_{(1)}]$ and define it to be our environmental constant. Thus we get Eq.(10):

$${\mathbb{E}}[{d}^{2}(t+1)| d(t)]={d}^{2}(t)+{r}^{2}-2rd{K}_{n,N}$$

From these limits, we can quantify the trade-off. The speed of adaptation far from the peak is found by analysing the average change in distance, Δ*d*. We can use the approximation ${\mathbb{E}}[d(t+1)]\approx \sqrt{d{(t)}^{2}-2{K}_{n,N}rd(t)}\approx d(t)-{K}_{n,N}r$ (See below). Thus, the speed of progression is proportional to *r*. The accuracy is determined by the equilibrium distance (the mutation-selection balance, d^*^), where the expected change is zero. From Eq. (10),we get that within the strong-selection approximation,

$$0={r}^{2}-2{K}_{n,N}r{d}^{* }\,\Rightarrow \,{d}^{* }=\frac{r}{2{K}_{n,N}}$$

Since both speed ( ∝ *r*) and inaccuracy ( ∝ *r*) scale with the mutation radius, a fixed-rate strategy cannot be both fast and accurate.

To calculate the speed of progression when a population is far away from the peak, *d* ≫ 1, we proceed as follows. Note

$${\mathbb{E}}[d(t+1)-d(t)| d(t)]\approx {\mathbb{E}}[\sqrt{{d}^{2}(t+1)}| d(t)]-d(t),$$

and so proceeding as when deriving Eq.(10), we get:

$${\mathbb{E}}[d(t+1)-d| d]={\mathbb{E}}[\sqrt{{({d}^{M})}_{(1)}^{2}(t+1)}| d]-d(t)={\mathbb{E}}[\sqrt{{(d-r)}^{2}+4rd{B}_{(1)}}| d]-d.$$

As such proceeding by Taylor expansion as *r*/*d* ≪ 1, we get:

$${\mathbb{E}}[d(t+1)-d(t)| d(t)]=d\left({\mathbb{E}}[1+\frac{{r}^{2}}{2{d}^{2}}-\frac{r}{d}+\frac{2r}{d}{B}_{(1)}-\frac{{(4{B}_{(1)}-2)}^{2}{r}^{2}}{8{d}^{2}}+{\mathcal{O}}({d}^{-3})]-1\right).$$

Thus simplifying we have

$${\mathbb{E}}[d(t+1)| d(t)]-d(t)=-r{K}_{n,N}+{\mathcal{O}}({d}^{-1}).$$

### Derivation of the optimal plastic strategy

A plastic strategy, *r*_opt_(*d*), can resolve this trade-off. We find it by choosing the value of *r* that minimizes ${\mathbb{E}}[{d}^{2}(t+1)| d(t)]$ at each distance *d*(*t*).

In the strong selection regime (*d* ≫ 1), we minimize Eq. (10) with respect to *r* by taking the derivative and setting it to zero:

$$\frac{\partial }{\partial r}\left({d}^{2}+{r}^{2}-2{K}_{n,N}rd\right)=2r-2{K}_{n,N}d=0$$

Solving for *r* gives the optimal strategy in this regime:

$${r}_{{\rm{opt}}}(d)\approx {K}_{n,N}d$$

In the weak selection regime (*d* ≪ 1), we analyse Eq. (11). As noted, for any *r* > 0, the expected squared distance increases. To minimize this deleterious effect, the optimal strategy is to have a minimal mutation rate:

$${r}_{{\rm{opt}}}(d)\approx 0$$

Together, these results show that the optimal strategy is biphasic: it scales linearly with maladaptation when far from the optimum and is minimal when close to it. The transition between these regimes occurs at a critical distance of *d* ≈ 1, as is argued below.

To understand how the two regimes are matched in the biphasic strategy, we analysed the general case, i.e., our starting point is Eq. (12). Recall that we defined *f*_*n*,*N*_(*r*, *d*) to be

$${f}_{n,N}(r,d)=\left(1-2{\mathbb{E}}\left[\mathop{\sum }\limits_{i=1}^{N}\frac{{B}_{i}\exp (-nrd{B}_{i})}{{\sum }_{k=1}^{N}\exp (-nrd{B}_{k})}| d\right]\right),$$

For us any optimal strategy *r*(*d*; *n*, *N*) will be one that minimises ${\mathbb{E}}[{d}^{2}(t+1)| d(t)].$ As such to find it we differentiate Eq.(9) to get:

$$\frac{\partial {\mathbb{E}}[{d}^{2}(t+1)| d(t)]}{\partial r}=2r-2d(t){f}_{n,N}(r,d(t))-2rd(t){g}_{n,N}(r,d(t)),$$

where

$${g}_{n,N}:=\frac{\partial {f}_{n,N}}{\partial r}=2nd{\mathbb{E}}\left[\frac{{\sum }_{i=1}^{N}{B}_{i}^{2}{e}^{-nrd{B}_{i}}}{{\sum }_{k=1}^{N}{e}^{-nrd{B}_{k}}}-\frac{{({\sum }_{i=1}^{N}{B}_{i}{e}^{-nrd{B}_{i}})}^{2}}{{({\sum }_{k=1}^{N}{e}^{-nrd{B}_{k}})}^{2}}\right].$$

For notational purposes, it will be good to note the following. Namely, that ${e}^{-nrd{B}_{i}}/\sum {e}^{-nrd{B}_{k}}$ can be seen as a “probability measure", namely a Boltzmann like distribution, with different *B*_*i*_ being different “energy states". We can gain intuition by defining a “pseudo-expectation", ${\mathcal{E}}$, and a “pseudo-variance", ${\mathcal{V}}$, in the natural way. Namely

$${\mathcal{E}}({\bf{B}};\alpha )=\frac{{\sum }_{i=1}^{N}{B}_{i}{e}^{-\alpha {B}_{i}}}{{\sum }_{k=1}^{N}{e}^{-\alpha {B}_{k}}},$$

where **B** refers to the vector with component *i* being given by *B*_*i*_, and we define ${\mathcal{V}}$ similarly. Thus we write,

$${f}_{n,N}(r,d)=1-2{\mathbb{E}}[{\mathcal{E}}({\bf{B}};nrd)],$$

and

$${g}_{n,N}=2nd{\mathbb{E}}\left[{\mathcal{V}}({\bf{B}};nrd)\right].$$

Therefore we can write our differential equation as

$$\frac{\partial {\mathbb{E}}[{d}^{2}(t+1)| d]}{\partial r}=2r-2d\left(1-2{\mathbb{E}}[{\mathcal{E}}({\bf{B}},nrd)]\right)-4rn{d}^{2}{\mathbb{E}}\left[{\mathcal{V}}({\bf{B}},nrd)\right].$$

Thus a minimising *r*(*d*; *n*, *N*) would satisfy

$$0=r-d\left(1-2{\mathbb{E}}[{\mathcal{E}}({\bf{B}},nrd)]\right)-2rn{d}^{2}{\mathbb{E}}\left[{\mathcal{V}}({\bf{B}},nrd)\right].$$

When *d* = 0, it is clear that the optimal mutation radius is *r* = 0, so suppose that *d* ≠ 0, and consider the scaling inspired by Eq.(10), namely *ω* = *r*/*dK*_*n*,*N*_. We get

$$0=\omega -\frac{\left(1-2{\mathbb{E}}[{\mathcal{E}}({\bf{B}},n{K}_{n,N}\omega {d}^{2})]\right)}{{K}_{n,N}}-2\omega n{d}^{2}{\mathbb{E}}\left[{\mathcal{V}}({\bf{B}},n{K}_{n,N}\omega {d}^{2})\right].$$

Rearranging, we see that

13 $$\omega \left(1-2n{d}^{2}{\mathbb{E}}\left[{\mathcal{V}}({\bf{B}},n{K}_{n,N}\omega {d}^{2})\right]\right)=\frac{1-2{\mathbb{E}}[{\mathcal{E}}({\bf{B}},n{K}_{n,N}\omega {d}^{2})]}{{K}_{n,N}},$$

and we see that *ω* = 0 is always a solution to this equation, as ${\mathcal{E}}({\bf{B}},0)$ corresponds to the case of equal weighting, and ${\mathbb{E}}[{B}_{i}]=1/2$. Note also that as *d*(*t*) → *∞* (keeping *ω* fixed), we get that the right hand side of Eq.(13) goes to 1, precisely by the same argument as when we derived Eq.(10), while the left hand side goes to *ω* as ${\mathcal{V}}({d}^{2}{\bf{B}},nK\omega )\to 0$ as *d* → *∞* To see this one can imagine *d*^2^*B*_*i*_ as the new variables, and note that the minimal one will be selected with probability one, in the limit of *d* → *∞*, as such the “variance" of this process will be 0, as it is a constant random variable. As such we recover *ω* = 1 in the limit *d* → *∞*, which is precisely the strong selection optimal strategy. Note also that close to the peak Eq. (11) tells us that *ω* = 0 is the only solution (One can also do a Taylor expansion of (13) to discover the same result). As such we expect there to be some transitional distance *d*_*Tr*_, at which point *ω*(*d*) = 0 for *d* ≤ *d*_*Tr*_ and *ω* > 0 for *d* > *d*_*Tr*_. To find it we expand the function

$$h(\omega )=\omega -\frac{\left(1-2{\mathbb{E}}[{\mathcal{E}}({\bf{B}},n{K}_{n,N}\omega {d}^{2})]\right)}{{K}_{n,N}}-2\omega n{d}^{2}{\mathbb{E}}\left[{\mathcal{V}}({\bf{B}},n{K}_{n,N}\omega {d}^{2})\right],$$

around *ω* = 0. We spare the details, noting that the expansion proceeds similarly to that in the derivation of Eq.(11). We get:

$$h(\omega )=\omega -\frac{1}{{K}_{n,N}}\frac{{K}_{n,N}{d}^{2}\omega (N-1)}{2N}-2\omega n{d}^{2}(t)\frac{N-1}{4nN}+{\mathcal{O}}({\omega }^{2}),$$

and thus simplifying we have

$$h(\omega )=\omega -\frac{{d}^{2}\omega (N-1)}{N}+{\mathcal{O}}({\omega }^{2}).$$

Thus once again under the mild assumption on the size of the population so that (*N* − 1)/*N* ≈ 1, we get:

14 $$h(\omega )\approx \omega -{d}^{2}\omega +{\mathcal{O}}({\omega }^{2}).$$

Eq.(14) tells us that *h*(*ω*) = 0 when *ω* = 0, same as Eq. (13). More importantly, it tells us that *h*(*ω*) ≈ 0 when *d* = 1, and so the linear coefficient of *ω* changes sign near *d* = 1, suggesting that the positive solution branch emerges at a threshold close to *d* = 1. As such we expect *d*_thresh_ ≈ 1 as after *d* > 1, one can achieve a balance between the linear term in *ω*, *ω*(1 − *d*^2^) and the higher order terms, ${\mathcal{O}}({\omega }^{2})$. Moreover, note that in the (*d*, *ω*) scale that we chose, the transition point *d*_thresh_ is independent of any environmental parameters (for mildly large population sizes). Also note that from this we can see that *ω* as the *maximal* solution of Eq.(13), is continuous, as for *d* < 1 that follows as *ω* is constant. Then after *d*_thresh_ > 0, we have that it is the positive root of Eq.(13) and so continuity follows from the continuity of the terms of the equation, and the transition around *d*_thresh_ is continuous as seen above as close to the transition point *ω* is close to 0.

### Implementation via growth rate sensing

Since cells can measure their own growth rate but not their phenotypic distance from the optimum, we now re-express the optimal mutational strategy as a function of growth rate, yielding a biologically implementable control law.

The optimal per-generation mutation rate, *ρ*, is obtained by substituting *r*_opt_ into the scaling relation $r=\sqrt{2\beta \rho }$:

$${\rho }_{{\rm{bph}}}(d)=\frac{{r}_{{\rm{opt}}}{(d)}^{2}}{2\beta }\approx \frac{{({K}_{n,N}d)}^{2}}{2\beta }{\mathcal{S}}(d)$$

where ${\mathcal{S}}(d)$ is a switch function that is ≈ 1 for *d* > 1 and ≈ 0 for *d* < 1. We use the relationship between growth rate and distance, $\mu (d)={\mu }_{\max }{e}^{-n{d}^{2}/4}$, to solve for *d*^2^:

$${d}^{2}=\frac{4}{n}\log \left(\frac{{\mu }_{\max }}{\mu }\right)$$

Substituting this into the expression for *ρ*_bph_(*d*) yields:

15 $${\rho }_{{\rm{bph}}}(\mu )\approx \frac{{K}_{n,N}^{2}}{2\beta }\left[\frac{4}{n}\log \left(\frac{{\mu }_{\max }}{\mu }\right)\right]{{\mathcal{S}}}_{\kappa }(\mu )$$

where ${{\mathcal{S}}}_{\kappa }(\mu )$ is the switch function expressed in terms of growth rate, that is *S*_*κ*_ ≈ 1 for *μ* < *κ* and *S*_*κ*_ ≈ 0 for *μ* > *κ*.

By grouping the constant terms, we can identify the expressions for the mutational gain, *α*, and the critical growth rate, *κ*.

- **Optimal Mutational Gain (*α*):** The constants multiplying the logarithm term define the optimal mutational gain:
  16 $$\alpha =\frac{2{K}_{n,N}^{2}}{n\beta }$$
- **Optimal Critical Growth Rate (*κ*):** The switch point occurs at the critical distance *d* = 1. We find the corresponding growth rate by evaluating *μ*(*d* = 1):
  17 $$\kappa =\mu (d=1)={\mu }_{\max }{e}^{-n{(1)}^{2}/4}={\mu }_{\max }{e}^{-n/4}$$

Substituting these definitions yields the final form presented in the main text:

$${\rho }_{{\rm{bph}}}(\mu )\approx \alpha \log \left(\frac{{\mu }_{\max }}{\mu }\right){{\mathcal{S}}}_{\kappa }\left(\mu \right).$$

### Timescale in the model

Our model is expressed in terms of generation number, so here we provide a way to relate the generational timescale to conventional time (e.g., minutes or hours). Suppose that within the population there exists a single individual whose growth rate is much higher than that of all others, i.e., *μ*_*m*_ ≫ *μ*_*i*_ for all *i* ∈ {1, …, *N*}{*m*}. In the corresponding strong-selection limit, the next Wright-Fisher generation is effectively descended from the individual with the highest growth rate. If each generation contains *N* individuals, it will therefore take *k* divisions for this lineage to repopulate the next generation, where 2^*k*^ = *N* (assuming, for simplicity, that *N* is a power of two). The corresponding generational timescale is then approximated by

$${t}_{{\rm{gen}}}\approx \frac{\log N}{{\mu }_{m}}.$$

Because the remaining individuals also grow, this represents an upper bound on the generational time,

$${t}_{{\rm{gen}}}\le \frac{\log N}{{\mu }_{m}},$$

with equality holding in the strong-selection limit.

### Selection in the model

In this section, we present a definition for selection strength *β*. Recall that our growth rate is given by

$$\mu ={\mu }_{\max }\exp (-\frac{1}{2}{(x-{x}^{* })}^{T}S(x-{x}^{* })),$$

where *S* is a positive semi-definite matrix.

Consider (without loss of generality) x^*^ = 0, and let *x*, *y* be two collinear phenotypes in ${{\mathbb{R}}}^{\widehat{n}}$, so that *γx* = *y*, where *γ* > 1, so that *y* is further away from the peak. The probability of selecting phenotype *x* is then,

$${{{\mathbb{P}}}}(x\,{\text{ is }}\,{\mathrm{selected}})=\frac{{e}^{-\frac{1}{2}{x}^{T}Sx}}{{e}^{-\frac{1}{2}{x}^{T}Sx}+{e}^{-\frac{1}{2}{\gamma }^{2}{x}^{T}Sx}}=\frac{1}{1+{e}^{-\frac{1}{2}({\gamma }^{2}-1){x}^{T}Sx}}.$$

So if we look at the direction in which selection is maximised, we get

$$\mathop{\max }\limits_{| | x| | =1}{{{\mathbb{P}}}}(x\,{\text{ is }}\,{\mathrm{selected}})=\frac{1}{1+{{\mathrm{min}}}_{| | x| | =1}{e}^{-\frac{1}{2}({\gamma }^{2}-1){x}^{T}Sx}}=\frac{1}{1+{e}^{-\frac{1}{2}({\gamma }^{2}-1)\,{{\mathrm{max}}}_{| | x| | =1}{x}^{T}Sx}},$$

as *γ* > 1. As *S* is positive semi-definite, and thus its eigenvalues are real and non-negative, this allows us to use the variational characterisation of the maximal eigenvalue to get,

$$\mathop{\max }\limits_{| | x| | =1}{{{\mathbb{P}}}}(x\,{\text{ is }}\,{\mathrm{selected}})=\frac{1}{1+{{\mathrm{min}}}_{| | x| | =1}{e}^{-\frac{1}{2}({\gamma }^{2}-1){x}^{T}Sx}}=\frac{1}{1+{e}^{-\frac{1}{2}({\gamma }^{2}-1){\lambda }_{\max }}},$$

where ${\lambda }_{\max }$ is the maximal eigenvalue of *S*. We thus define the selection strength to be $\beta :={\lambda }_{\max }$, the largest eigenvalue of *S*. This quantity gives the maximal curvature of the log-growth landscape and thus sets the strongest scale of stabilizing selection across phenotypic directions. Note that when *β* = 0, ${\mathbb{P}}(x\,{\text{ is}\,{selected}})=1/2$, in this pairwise comparison, and as *β* → *∞*, $\mathop{\max }\nolimits_{| | x| | =1}{\mathbb{P}}(x\,{\text{ is}\,{selected}})\to 1$, i.e., along the direction of the maximal eigenvalue, selection is strong (the individual with the higher growth rate always gets selected).

Now let us look at the effect of selection on the number of distinct phenotypes in the population. Let *p*_*i*_ be the probability of phenotype *i* being selected (after mutation). Then the probability, that a phenotype *i* disappears is, ${\mathbb{P}}({\text{phenotype }}\,i\,{\text{ disappears}})={(1-{p}_{i})}^{N}$. Therefore after selection we have

$${\mathbb{E}}[\#\,{\mathrm{phenotypes}}]=\mathop{\sum }\limits_{i}{\mathbb{E}}[{\text{phenotype }}\,i\,{\text{ is}}\,{\text{ still}}\,{\text{ present}}],$$

and so

$${\mathbb{E}}[\#\,{\mathrm{phenotypes}}]=\mathop{\sum }\limits_{i}(1-{(1-{p}_{i})}^{N}) \sim N-\sum {e}^{-N{p}_{i}},$$

where we take the regime of a large population size and *p*_*i*_ small.

### Behaviour of the environmental constant

In this section, we examine the environmental constant, *K*_*n*,*N*_, and provide asymptotic behaviour for it and an intuition as to how it behaves generally. Mathematically, we have an exact formula for *K*_*n*,*N*_, namely

18 $${K}_{n,N}=1-2{\int }_{0}^{1}{I}_{x}{(\nu ,\nu )}^{N}dx,$$

where *I*_*x*_(*a*, *b*) is the regularised incomplete beta function and we set *ν* ≔ (*n* − 1)/2 for simplicity. *K*_*n*,*N*_ is close to 1 for low *n*, and high *N*; and close to 0 for high *n* and low *N*. This is precisely in accordance with the fact that *K*_*n*,*N*_ is a measure of the “ease of adaptation", the lower the phenotypic dimension, the easier it is for adaptation to occur, and the higher the population size, the more likely it is for a beneficial adaptation to occur and fixate in a population.

We derive an asymptotic approximation for Eq.(18). To do this, we turn to extreme value theory. Firstly, recall that

$${\int }_{0}^{1}{I}_{x}{(\nu ,\nu )}^{N}dx={\mathbb{E}}[{B}_{(1)}],$$

where *B*_*i*_ ~*i*. *i*. *d*Beta(*ν*, *ν*), and the subscript (1) refers to the minimal value over *N* random variables. As such, we can employ results from extreme value theory, which concerns the distribution of the maximal and minimal values of independent random variables.

Now extreme value theory tells us that

$${\rm{{\mathbb{P}}}}\left({B}_{(1)}\le x{\left(\frac{\nu B(\nu ,\nu )}{N}\right)}^{1/\nu }\right)\to 1-{e}^{-{x}^{\nu }},$$

as *N* → *∞*. Recall that as *B*_(1)_ is a positive random variable, we can calculate its expectation as

$${\mathbb{E}}[{B}_{(1)}]={\int }_{0}^{1}{\mathbb{P}}({B}_{(1)}\ge x)dx.$$

By a substitution of *x* = *u*(*νB*(*ν*, *ν*)/*N*)^1/*ν*^, we get

$${\mathbb{E}}[{B}_{(1)}]=c{\int }_{0}^{1/c}{\rm{{\mathbb{P}}}}\left({B}_{(1)}\ge x{\left(\frac{\nu B(\nu ,\nu )}{N}\right)}^{1/\nu }\right)dx,$$

where *c* = (*νB*(*ν*, *ν*)/*N*)^1/*ν*^, so assuming *N* is large, we can approximate the integrand using extreme value theory as above, to get

$${\mathbb{E}}[{B}_{(1)}]\approx c{\int }_{0}^{1/c}{e}^{-{x}^{\nu }}dx\approx c{\int }_{0}^{\infty }{e}^{-{x}^{\nu }}dx,$$

where in the second approximation once again we utilise a large population size, and also the fact that most of the mass of the integrand is in the interval (0, 1). Now this integral is just a Gamma function in disguise, as the substitution *z* = *x*^*ν*^ reveals, and we get

$${\mathbb{E}}[{B}_{(1)}]\approx \frac{c}{\nu }\Gamma \left(\frac{1}{\nu }\right),$$

and so our environmental constant is given by

$${K}_{n,N}\approx 1-\frac{2\Gamma (1/\nu )}{\nu }{\left(\frac{\nu B(\nu ,\nu )}{N}\right)}^{1/\nu }.$$

This approximation is good for medium values of *n* and large *N*. For a more intuitive form, we may apply Stirling’s approximation to get that *K*_*n*,*N*_ behaves approximately like

$${K}_{n,N}\approx 1-\frac{1}{2{N}^{1/\nu }}.$$

Thus for relatively large population sizes (*N* ≿ 100), the value of the environmental constant remains approximately unchanged. Thus, we see that *K*_*n*,*N*_ is robust to perturbations in population sizes, which are not exponential in nature. This, in turn, allows the strategies we presented above to be robust to most fluctuating colony sizes.

An exact analytical solution can be obtained in the cases of a low population size, *N* = 2, *N* = 3, and we have that

$${K}_{n,2}=\frac{2B(2\nu ,2\nu )}{\nu {[B(\nu ,\nu )]}^{2}},{K}_{n,3}=\frac{3}{2}K(n,2)$$

where *B*(*a*, *b*) is the beta function. If we denote the integral as

19 $$J(\nu ,N):={\int }_{0}^{1}{I}_{x}{(\nu ,\nu )}^{N}dx,$$

there are a few properties that can be deduced about *J*. Firstly, note that as *I*_1−*x*_(*a*, *b*) = 1 − *I*_*x*_(*b*, *a*), we have that

$$J(\nu ,N)={\int }_{0}^{1}{(1-{I}_{u}(\nu ,\nu ))}^{N}du.$$

As such we have the following relations between the *J*s:

20 $$J(\nu ,N)=\mathop{\sum }\limits_{k=0}^{N}{(-1)}^{k}\left(\begin{array}{c}N\\ k\end{array}\right)J(\nu ,k).$$

The derivation utilises this fact, as if we know the values of *J*(*ν*, *k*) when *k* is even, up to some *N* = 2*m*, we can determine the values of *J*(*ν*, *k*) for odd *k* up to *N* = 2*m* + 1. For the sake of brevity we do not provide the full details of the derivation here.

We also note that for large *n* by means of a Stirling approximation, we have

$${K}_{n,2} \sim \frac{1}{\sqrt{2\pi \nu }},{K}_{n,3} \sim \frac{3}{2\sqrt{2\pi \nu }},$$

Thus for a low population size, the environmental constant decays like $1/\sqrt{n}$.

### Fluctuations in the critical growth rate and the anchoring heuristic

In the main text, we only examined phenotypic fluctuations in the mutational gain *α*. In this section, we examine how fluctuations in *κ* also influence the adaptation process in fluctuating environments.

Phenotypic fluctuations in the critical growth rate, *κ*, also aid in adaptation in a single environment setting (Fig. S4), as opposed to phenotypic fluctuations in the mutational gain, *α*, only (Fig. 4). This suggests that the additional adaptation is driven by fluctuations in *κ*. The reason for this is the following; when *κ* fluctuates, individuals that stochastically receive a higher *κ* continue mutating closer to the peak than the population average (recall that higher *κ* corresponds to lower threshold distance), increasing the average *κ* threshold progressively. This allows the population to continue adapting beyond the point at which a fixed-threshold strategy would typically stall, while preserving much of the initial adaptation speed.

This mechanism can be illustrated by ignoring phenotypic fluctuations themselves and instead considering a deterministic heuristic strategy (in the sense that the parameters of the strategy are fixed), which we call the anchoring strategy. Let ${d}_{\min }$ be $\min \{{d}_{1},...,{d}_{N}\}$, the minimal phenotypic distance to the peak of the most adapted individual. Define the anchoring strategy as follows: for an individual with a phenotypic distance *d*_*i*_ its mutational radius *r*_*i*_ is given by

$${r}_{i}({d}_{i})={K}_{n,N}{d}_{i}H({d}_{i};\min \{{d}_{\min },1\}),$$

where *H*(*x*; *y*) is the Heaviside function with the jump at *y*, i.e., *H*(*x*, *y*) = 1 for *x* > *y* and *H*(*x*, *y*) = 0 for *x* < *y*. As such whenever all of *d*_*i*_ > 1, the anchoring strategy is equivalent to the biphasic one, but when one of the *d*_*i*_ < 1, the strategy is such that the individual closest to the peak doesn’t mutate (i.e., its phenotype is anchored), while the rest of the individuals mutate at an optimal rate. The anchoring strategy systematically exploits population variance: the fittest individual in the population acts as an anchor with a zero mutation rate, preserving the lowest attained phenotypic distance without incurring mutational load. Concurrently, the remainder of the population maintains *r* > 0, continuously exploring the phenotypic space until a novel variant with a lower distance emerges to replace the anchor. While the initial mean rate of adaptation is comparable to a standard linear strategy, this anchoring mechanism sustains greater phenotypic variance and allows the population’s mean distance to converge arbitrarily close to the optimal peak (Figure S3).

The anchoring strategy is in some sense the “best case outcome” of fluctuations in the critical growth rate, *κ*, namely the higher growth phenotypes do not exhibit mutagenesis, and so stay fixed, while the lower growth phenotypes exhibit mutagenesis and so can potentially find a mutation to improve their own growth rate, when compared to the higher growth phenotypes, thus explaining the further adaptive potential gained from having phenotypic fluctuations in *κ*.

### Simulation details

All the simulations were performed in Python utilising the numpy, scipy and matplotlib packages.

We implemented the model as described in the Model Details section of the Methods. At the start of the simulations all individuals began with the same phenotype at a prescribed distance to the optimum, *d*_0_. Then to determine the next generation individuals were mutated (according to the corresponding strategies or lack thereof) and then selected via the Wright-Fisher procedure as described above. Parameters like the mutational gain or critical growth rate were modelled as being inherited by the offspring. When phenotypic fluctuations are present in the simulations, we sampled the mutational gains and the critical growth rates from a Uniform distribution over an interval with the probability of a phenotypic change in these parameters occurring in an individual within a generation being a prescribed probability *p*. In the simulations comparing the population with no phenotypic fluctuations and a population with fluctuations, both populations start with variability in the population (i.e., *α*_*i*_(0) ~ Unif(0, 1) and if applicable *κ*_*i*_(0) ~ Unif(0, 5)).

Figure 3 is generated in the following way. We first select *β*_1_, *β*_2_ values for two different environments (In the figure *β*_1_ = 1, *β*_2_ = 2). We then calculate the optimal mutational gain in both environments *s*_1_, *s*_2_ according to equations presented in the Methods above. This gives the optimal strategies in both environments, *r*_1_, *r*_2_ respectively.

To generate Fig. 3B, we transform the strategies according to Eqs.((15),(16),(17)). Note *α*_*i*_ = 2*s*_*i*_/*nβ*. Note that when the environment shifts the mutation rate of the optimal strategy in the first environment, *ρ*_1_, remains constant, but the **mutational radius changes** and we have ${r}_{1,{\rm{Env.}}2}=\sqrt{2{\beta }_{2}{\rho }_{1}}$.

The shift in *β* between environments effectively scales the mutational radius by $\sqrt{{\beta }_{2}/{\beta }_{1}}$. So ${r}_{1,{\rm{Env.}}2}=\sqrt{({\beta }_{2}/{\beta }_{1})}{K}_{n,N}{d}_{{\rm{thresh}}}.$Note *d* depends on the growth rate, maximal growth rate and the number of phenotypic dimensions. As such the threshold *d*_thresh_ doesn’t change between environmental shifts. Furthermore, during the environmental shift in Fig. 3, we reset the phenotypic distance to *d*_0_ (*d*_0_ = 2.5 in the Figure).

## Supplementary information

Supplementary Information

## Data Availability

This study did not generate new experimental datasets. All results presented in this manuscript can be reproduced using the simulation code available at https://github.com/karin-lab/mutation_rate_2026/. Parameters used for each simulation are provided in the corresponding figure captions and in the Methods section.

## Code availability

All code used to generate the simulations and figures is available at https://github.com/karin-lab/mutation_rate_2026/.
