# Supplementary material for: Growth rate-dependent mutations and phenotypic switching facilitate rapid adaptation
Source: NPJ Syst Biol Appl. 2026 Jun 16;12:146. doi: 10.1038/s41540-026-00758-4 (PMC13635105; doi:10.1038/s41540-026-00758-4)
Supplement: Supplementary file 1 [file 41540_2026_758_MOESM1_ESM.pdf]

Supplemental Material for *Growth rate-dependent mutations and phenotypic switching facilitate rapid adaptation*

Supplementary Figures and Tables

Model parameter	Model interpretation	Biological interpretation (in particular in *E. coli*)	Plausible Molecular mechanisms (in *E. coli*)
n	Effective number of phenotypic dimensions (i.e. rank of the selection matrix S)	Number of independent traits under selection in a given environment, where independence is meant in a linear algebraic sense.	Environmental factor, dependent on which organismal traits are relevant for growth in that environment.
$\mu_{\max}$	Maximum possible growth rate (fitness) in a given environment.	Highest achievable growth rate for a given environment (e.g. for a given temperature). log(2)/(fastest doubling time)	Determined by many physiological and environmental factors, including nutrient availability, translational capacity, and metabolic efficiency.
d	Phenotypic distance to the optimal phenotype (i.e. one with $\mu_{\max}$).	Proxy measure of how well-adapted an individual is to the environment ($d = 0$ perfect adaptation). Not directly measurable as a molecular quantity. Under the assumption in the paper that growth rate is the only "fitness variable" of importance, it is simply a rescaling of the individual growth rate.	Not directly measurable as a molecular quantity.
$\mu(d)$	Fitness of an individual. We take it with respect to phenotypic distance to be single peaked with Gaussian decay: $\mu(d) = \mu_{\max} e^{-nd^2/4}$	Growth rate (e.g. hr^{-1}). $\mu = \log(2)/T_d$, where T_d is the doubling time.	Determined by many physiological and environmental factors, including nutrient availability, translational capacity, metabolic efficiency, and stress burden.
β	Selection strength (maximal eigenvalue of S).	Measure of how strongly the environment penalizes phenotypic deviations from the optimum. ($\beta = 0$ neutral selection, $\beta \to \infty$ only the fittest phenotype survives).	Determined by the environment and by how strongly growth depends on deviations in the relevant phenotypic traits.
ρ	Expected number of mutations per genome per generation.	Genomic mutation rate; expected number of mutations in an individual per generation.	Both influenced by the environment and the organism. I.e. presence of mutagenic species in environment (e.g reactive oxygen species); and repair fidelity of DNA repair mechanisms (e.g. efficacy of mismatch repair, and recruitment/ non-recruitment of error-prone DNA polymerases).
r	Mutational radius, $r = \sqrt{2\rho\beta}$.	"Dimensionless strength" of mutation in a given environment, i.e. how far the phenotype moves under mutation in our phenotypic space.	See ρ above.

α	Mutational gain, i.e. strength of the biphasic response ($\rho_{\text{bph}} = \alpha \log(\mu_{\max}/\mu) S_\kappa(\mu)$).	The increase in mutagenesis as the logarithm of the growth rate decreases.	There are to possibilities. If mutation rate is constant per time unit, then mutational gain may be naturally interpreted as due to increasing time between generations in lower growth rate. Otherwise, mutations may be constant per generation, if, for example, they occur only during DNA replication. In such a case, we may expect that mechanisms such as changes in expression or availability of repair enzymes in lower growth rates may drive the corresponding increase in α.
κ	Critical growth rate, below which we have elevated mutagenesis in the biphasic response.	Growth-rate threshold below which elevated mutagenesis is activated, or equivalently above which mutagenic damage is more effectively controlled.	In general a multitude of plausible biological mechanisms (See also p). The main hallmark is a critical growth rate under which repair capacity decreases. Under general stress response, it could correspond to the growth rate below which RpoS begins to be recruited rapidly; which would also correspond to the growth rate below which recruitment of error prone DNA polymerases occurs. In oxidative stress may correspond to the point at which repair capacity, such as base excision repair, becomes insufficient relative to damage load.
N	Population size. (Fixed in this paper)	Effective population size	Determined primarily by ecological conditions, such as resource availability.
$K_{n,N}$	Environmental constant, such that the optimal mutational radius in an environment is $K_{n,N} d$	"Ease of adaptation" in a given environment. Measure of the expected directional gain toward the optimum in the strong-selection regime; larger $K_{n,N}$ means adaptation is easier because the best available mutation is, on average, more effective.	Determined from both the effective phenotypic dimensionality of the environment and the population size.
p	Phenotypic switching probability. In the main paper only α values can switch, while here in the supplementary both α and κ may switch.	Rate at which organisms switch their mutagenic response, such as due to long-term fluctuations in gene expression. Changes in the mutation rates here are presumed to be non-genetic.	Consider mutational gain switching probability, p_α and critical growth rate switching probability p_κ separately. Under general stress response, p_α would correspond to the rate of stochastic fluctuations in sigma factor activity. p_κ on the other hand could be influenced by stochastic fluctuations in regulators of DNA damage or stress responses, such as DNA repair regulators, as for example the cell-to-cell variation in Ada abundance.

τ	Number of generations between environmental switches.	Timescale of environmental changes.	Environmentally determined factor. However, note in our simulations τ is of the units of generations, and so if τ is constant (w.r.t. time), one needs to rescale as then there will be different number of generations in each environment (See A.1).

Table S1: Model parameters and their biological interpretation with plausible molecular mechanisms (when applicable).

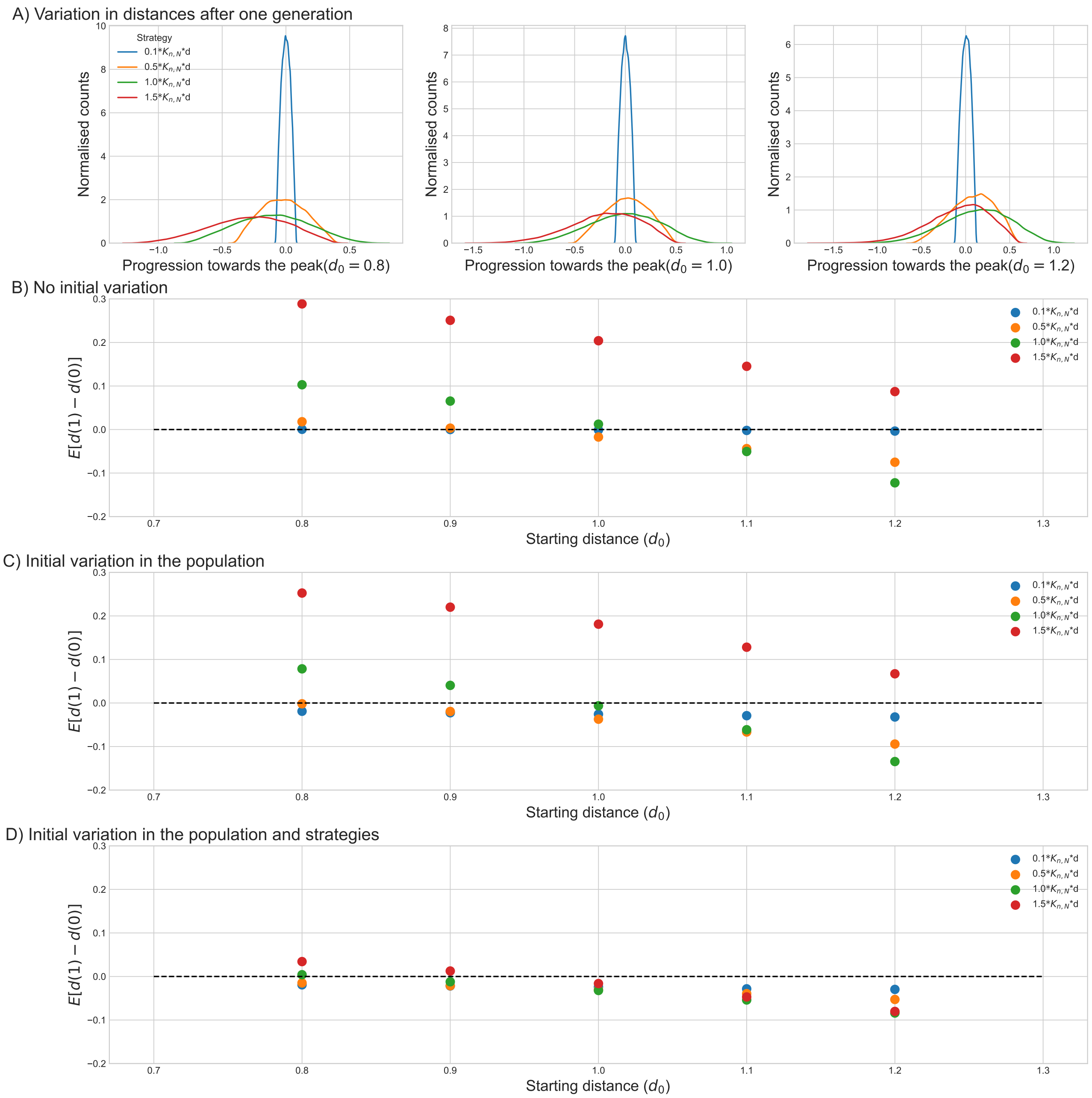

Figure S1: Variation in the weak selection regime contributes to adaptation. (A) The distribution of distances from optimal phenotype of the population after one generation (i.e. mutation and selection applied) when starting from a population with no variation. Three starting distances were used, from left to right, $d_0 = 1.2, d_0 = 1, d_0 = 0.8$. **(B,C,D)** The average improvement (/deterioration) in one generation of the distance to the optimum given a starting distance. (Improvement corresponding to negative y coordinate, as the distance is decreasing). Each point represents the performance of a given strategy. **(B)** A population with no initial variation following the $r = Cd$ strategy. **(C)** A population with initial variation following the $r = Cd$ strategy. **(D)** A population with initial variation and with variable strategies $r = U_iCd$ strategy, where $U_i \sim Unif(0, 1)$. Simulation parameters: Population size $N = 100000$, phenotypic dimensions $n = 5$

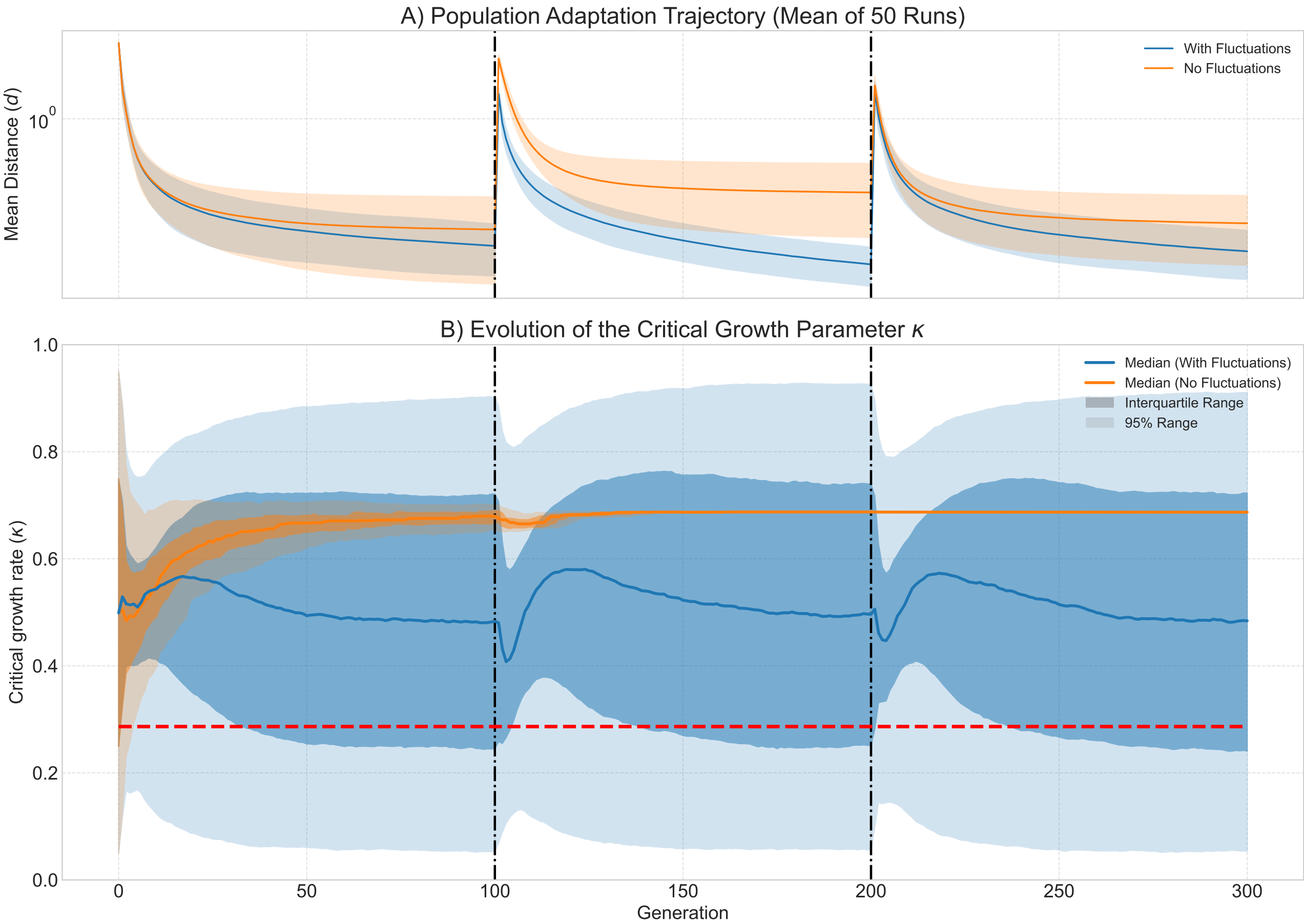

Figure S2: Phenotypic fluctuations maintain strategic diversity and facilitate rapid adaptation to novel environments. This figure, as Figure 3, compares the evolutionary dynamics of two populations over 300 generations, averaged over 50 independent simulations. Both populations evolve in a fluctuating environment that shifts at generations 100 and 200, with the selection strength changing from $\beta = 4.0$ (left) to $\beta = 0.5$ (centre) to $\beta = 2.0$ (right) One population experiences ongoing phenotypic fluctuations in its strategy parameters (blue), while the other does not (orange). **(A)** The mean population adaptation trajectory. The y-axis shows the mean phenotypic distance (d) from the optimum. The population with fluctuations consistently re-adapts faster following both environmental shifts. Shaded areas represent one standard deviation across the 50 simulation runs. **(B)** The evolution of the critical growth rate parameter, κ. The median evolved κ for each population is shown as a solid line, with layered shading representing the interquartile (darker shade) and 95% (lighter shade) ranges of the population's diversity. The red dashed line indicates the theoretical optimum for each environment (as predicted by the biphasic strategy). Simulation parameters: Population size $N = 5000$, phenotypic dimensions $n = 5$, fluctuation probability $p = 0.05$, and initial distance $d_0 = 2.5$ after each environmental shift. Initially both populations started with varied mutational gain and critical growth rate, with $\alpha_i \sim \text{Unif}(0, 1)$ and $\kappa_i \sim \text{Unif}(0, 1)$. In the population with fluctuations, the mutational parameters had a probability $p = 0.05$ of switching per individual per generation. If an individual switches its phenotype, the mutational gain was resampled uniformly from $(0, 1)$ (note $0 \leq K_{n,N} \leq 1$), and the critical threshold was resampled uniformly from $(0, 1)$ also.

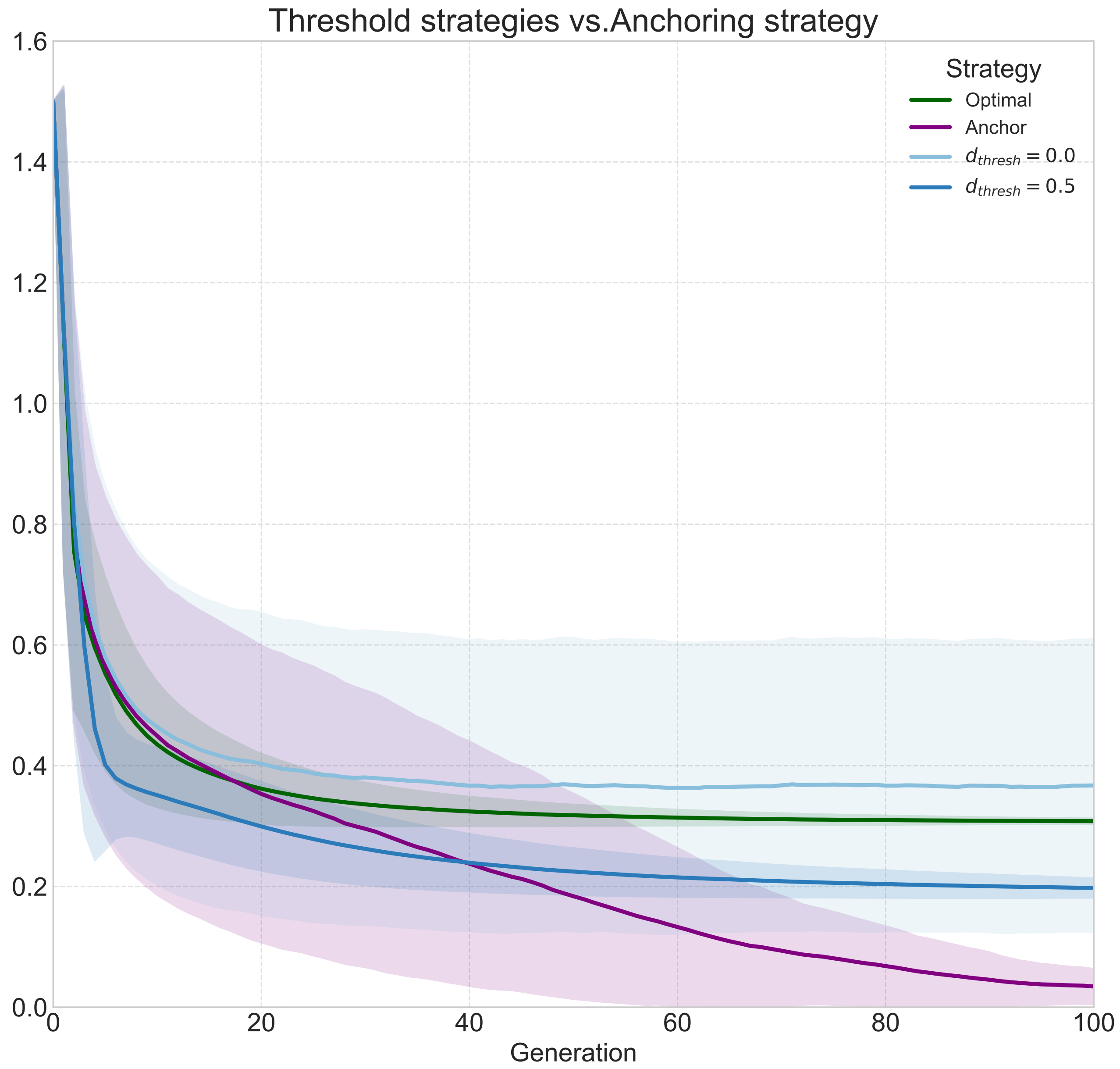

Figure S3: Comparison of the progression to the peak of the "anchoring" heuristic to the threshold strategies. Performance of various mutational strategies over 100 generations, starting from a phenotypic distance $d = 2.5$. Bold lines represent the mean of 50 replicate simulations, with the faint area being ± 1 standard deviation. The y-axis is on a logarithmic scale to highlight differences in equilibrium distance. The anchoring strategy is implemented by setting individual mutational radii to $r_i = K_{n,N} d_i$ if the individual distance is above $d = 1$ (i.e. $d_i > 1$, akin to the optimal strategy), or if there is an individual with distance closer to the peak (i.e. $d_i > d_{\min}$). Thus there is always an "anchor" at the best current phenotype.

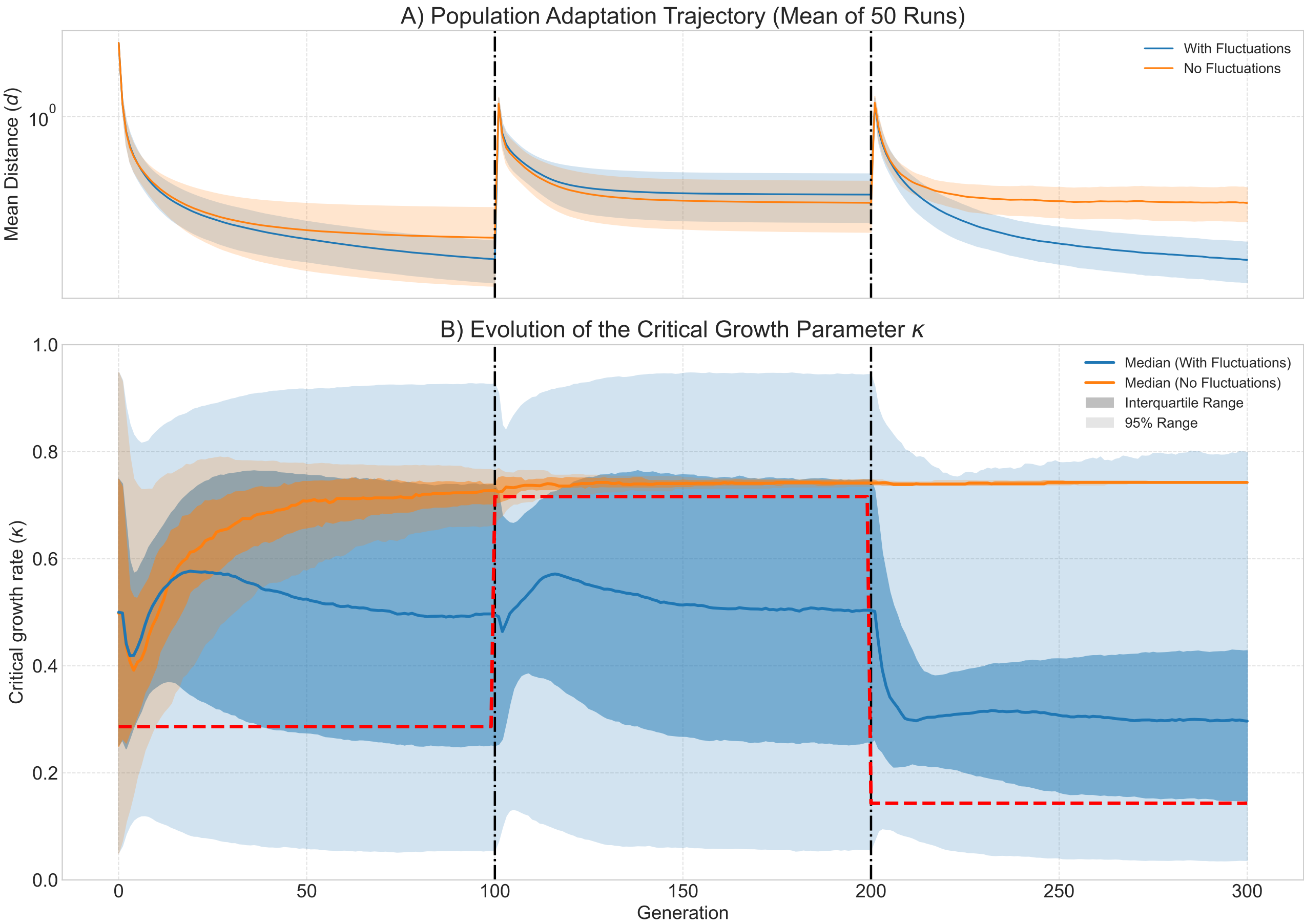

Figure S4: Phenotypic fluctuations maintain strategic diversity and facilitate rapid adaptation to novel environments. This figure, as Figure 3, compares the evolutionary dynamics of two populations over 300 generations, averaged over 50 independent simulations. Both populations evolve in a fluctuating environment that shifts at generations 100 and 200, with the maximal growth rate changing from $\mu_{\max} = 1.0$ (left) to $\mu_{\max} = 2.5$ (centre) to $\mu_{max} = 0.5$ (right) One population experiences ongoing phenotypic fluctuations in its strategy parameters (blue), while the other does not (orange). **(A)** The mean population adaptation trajectory. The y-axis shows the mean phenotypic distance (d) from the optimum. The population with fluctuations consistently re-adapts faster following both environmental shifts. Shaded areas represent one standard deviation across the 50 simulation runs. **(B)** The evolution of the critical growth rate parameter, κ. The median evolved κ for each population is shown as a solid line, with layered shading representing the interquartile (darker shade) and 95% (lighter shade) ranges of the population's diversity. The red dashed line indicates the theoretical optimum for each environment (as predicted by the biphasic strategy). Simulation parameters: Population size $N = 5000$, phenotypic dimensions $n = 5$, fluctuation probability $p = 0.05$, and initial distance $d_0 = 2.5$ after each environmental shift. Initially both populations started with varied critical growth rate, with $\kappa_i \sim \text{Unif}(0, 1)$. In the population with fluctuations, the mutational parameters had a probability $p = 0.05$ of switching per individual per generation. If an individual switches its phenotype, the critical threshold was resampled uniformly from $(0, 1)$ also.
